# Experimental and computational studies on a protonated 2-pyridinyl moiety and its switchable effect for the design of thermolytic devices

**DOI:** 10.1371/journal.pone.0203604

**Published:** 2018-09-20

**Authors:** Jolanta Brzezinska, Jacek Kujawski, Agnieszka Witkowska, Kornelia Czaja, Marek K. Bernard, Marcin K. Chmielewski

**Affiliations:** 1 Institute of Bioorganic Chemistry, Polish Academy of Sciences, Poznań, Poland; 2 Department of Organic Chemistry, Faculty of Pharmacy, Poznań University of Medical Sciences, Poznań, Poland; Duke University, UNITED STATES

## Abstract

1D and 2D NMR investigations as well as computational studies, including static quantum-mechanics calculations, density function theory formalism, and classical molecular dynamics, were applied to determine the protonation sites in the thermolabile protecting group (TPG) containing a 2-pyridynyl moiety within its structure. This protecting group has three possible sites for protonation: an azomethine (pyridinic) atom (*N1*), 2-aminoethanol residue (*N2*), and 4-amino substituent (*N4*). Our investigations showed that the protonation mainly occurs on the *N1* atom. Such protonation seems to be a major inhibitory factor in the thermal removal of 2-pyridynyl TPG by the “chemical switch” approach and decreases the aromaticity of the pyridine ring. We also discussed possible participation of *N*2 nitrogen in irreversible intramolecular cyclization under acidic conditions.

## Introduction

Reversible processes require that a substrate is restored from the product without any changes in its thermodynamic properties [1]. Within reversible processes we can distinguish spontaneous and enforced processes. A spontaneous reverse process occurs over an infinitesimally small time period, and the substrate and product are in equilibrium with each other [2]. However, many current approaches enforce the reversibility of processes by assembling two opposing irreversible processes. There are many methods for molecular coupling but the number of decoupling methodologies linked with them is rather limited. In this context, Coupling-and-Decoupling (CAD) Chemistry represents a new trend in the assembly of opposing chemical processes [3]. The assumption behind this approach is that reversibility should be as simple as possible to comply with. However, the reactivity of compounds can be a limiting factor and the reversible process has to be triggered to recreate the desired properties of a molecule. Currently, many useful applications are based on chemical linkers which connect two molecular units [4]. An example of reversibility in linker technology is cleavage of synthesized oligomers or polymers from solid supports [5]. A cleavable linker is suitable only for a specific application and its decoupling condition has to be designed for a target molecule (acid-, base-, photo-, enzymatic-labile linkers) [6]. The usefulness of *click* and *declick* reactions between amine and thiol groups with the use of a derivative of Meldrum's acid has been shown in protein chemistry [7]. The reversible chemical blocking strategy for RNA functionality is fulfilled by the reaction of a 2′-hydroxy group in aqueous buffer with an azide-substituted acylating agent (NAI-N_3_). Polyacylation (“cloaking”) can block RNA folding and/or hybridization. The subsequent removal of these groups (“uncloaking”) by phosphine treatment restores the RNA biophysical and biochemical activity[8].

Based on the above considerations, we developed two systems with reversible processes: “*click-clack*” (intramolecular cyclization and decyclization) [9] and “*chemical switch*” (transformation of a nitro into an amine group) [10] and demonstrated their usefulness in the regulation of thermolytic deprotection rates of 2-Pyridinyl Thermolabile Protecting Groups (2PyTPGs) [11]. The use of chemical modification to enable switching properties of 2PyTPGs increases the protecting group’s attractiveness.

Switchable reactions may be initiated either chemically or enzymatically [12] however, the most useful method is based on protonation, because it can be performed under mild and non-hazardous conditions [13]. Recently, we have shown the advantage of 2PyTPG as a perfect thermocontrolled group cleaved from the hydroxyl [14] and phosphate functions [15] in nucleosides and nucleotide chemistry [11]. Moreover, protonation of 2PyTPGs can be enabled by controlling their thermolabile properties [10]. We also proved that we were able to control this deprotecting process via intramolecular cyclization and modulation of the four factors: i) temperature, ii) electron distribution in the pyridine ring by various substituents on 4 position, iii) pH levels [16], and iv) steric effect [17].

Protection of the 5′-hydroxy group in nucleosides with carbonates of 2PyTPGs is reversible and can be modulated by changing those four factors. Nucleophilicity of pyridinic nitrogen is the driving force behind the thermocyclization process initiated by an attack of endocyclic nitrogen on the α-methylene bridge in the aminoethanol linker of potential 2PyTPG (S1 Fig). Therefore, through temperature or pH adjustments, we were able to control the rate of the intramolecular cyclization via protonation of *N*1 nitrogen [10] Although pyridinic nitrogen *N*1 participates in thermocyclization and its nucleophilicity determines this process, the role of other nitrogens, i.e. *N*2 from the aminoethanol substituent or *N4* from the 4-amino group, should not be neglected. Nitrogen *N*2 is involved in the cyclization of the 2PyTPG group and, together with the participation of the phosphate center, forms an oxazaphospholidine ring [18] Furthermore, *N*2 serves an important role in the oxidation of the H-phosphonate diester by an iodinated intermediate [15].

**Fig 1:**
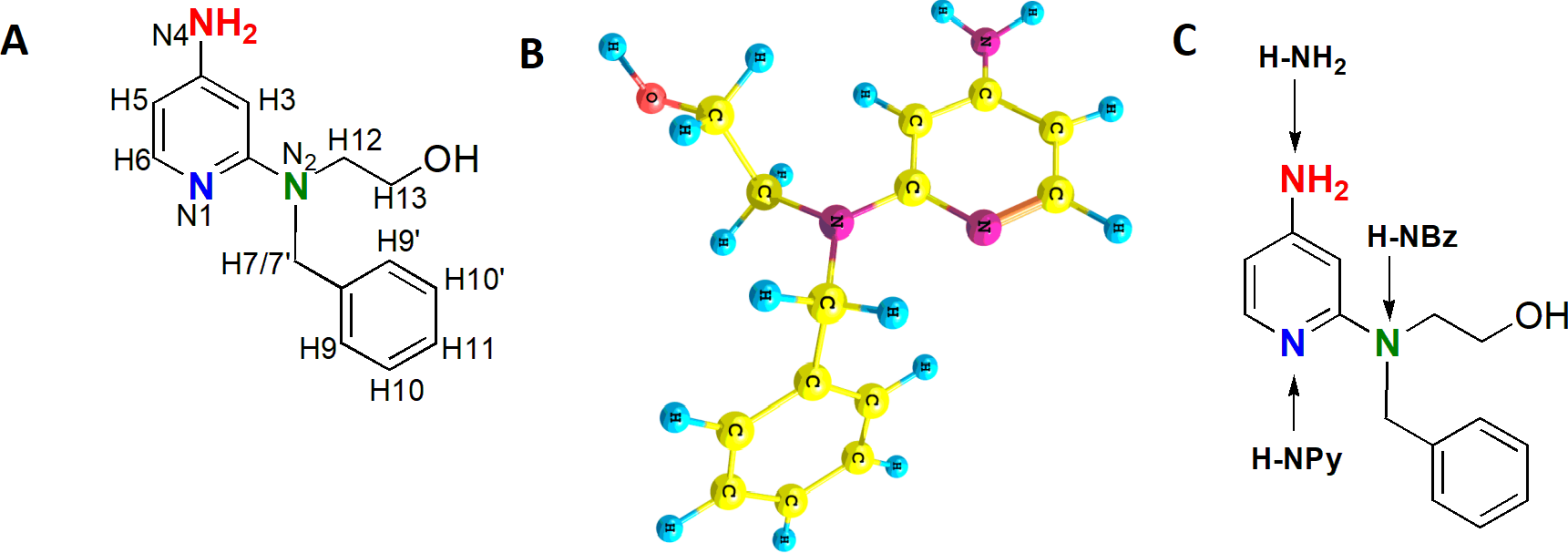
A: atom numbering for **1**: B: its rotamer (**I**); C: possible protonation sites for **1**.

In the present study, we demonstrate a correlation between NMR and computational studies of the protonation process for a representative of the 2-pyridinyl thermolytic devices. Thermocyclization is possible for the carbonate or phosphate forms of this protection group. However, in studies on proton affinity and the energy differences involving *N*1, *N*2, *N*4 protonation phenomena, we chose an amino alcohol, 4-amino-2-pyridinyl-*N*-benzylaminoethanol (**1**, *thermolytic precursor)*, because there is no difference between the carbonate and aminoalcohol protonations. There are three possible sites of protonation: a) endocyclic nitrogen (*N*1), b) 4-amino group (*N*4), and c) 2-amino group (*N*2) (Fig 1).

Nitrogen is an efficient atom for the expression of different hybridization states because of protonation or prototropic tautomerism, and ^15^N NMR is an excellent method for its detection [19].

## Results

### Assignment of proton, nitrogen and carbon atoms in compound 1

A complete assignment of the NMR signals of compound **1** (Fig 1) was performed using ^1^H, ^13^C, ^1^H-^1^H COSY, ^1^H-^13^C, ^1^H-^15^N HSQC/HMBC spectra. All spectra were recorded at 20°C in anhydrous DMSO-*d*_*6*_. The resonance assignments are presented in S1–S9 Tables.

In the ^1^H NMR spectrum, pyridine proton **H6** is observed at 7.58 ppm as a doublet coupled to **H5**. Both **H5** and **H3** signals are shifted upfield because of the electron donating effect of the 4-amino group. Proton **H5** appears as a doublet of doublets due to the coupling with **H6** and **H3**. The ethylene protons **H12** and **H13** are observed at 3.49 and 3.54 ppm, respectively. The amino group signal is present at 5.63 ppm as a sharp singlet.

**Fig 2:**
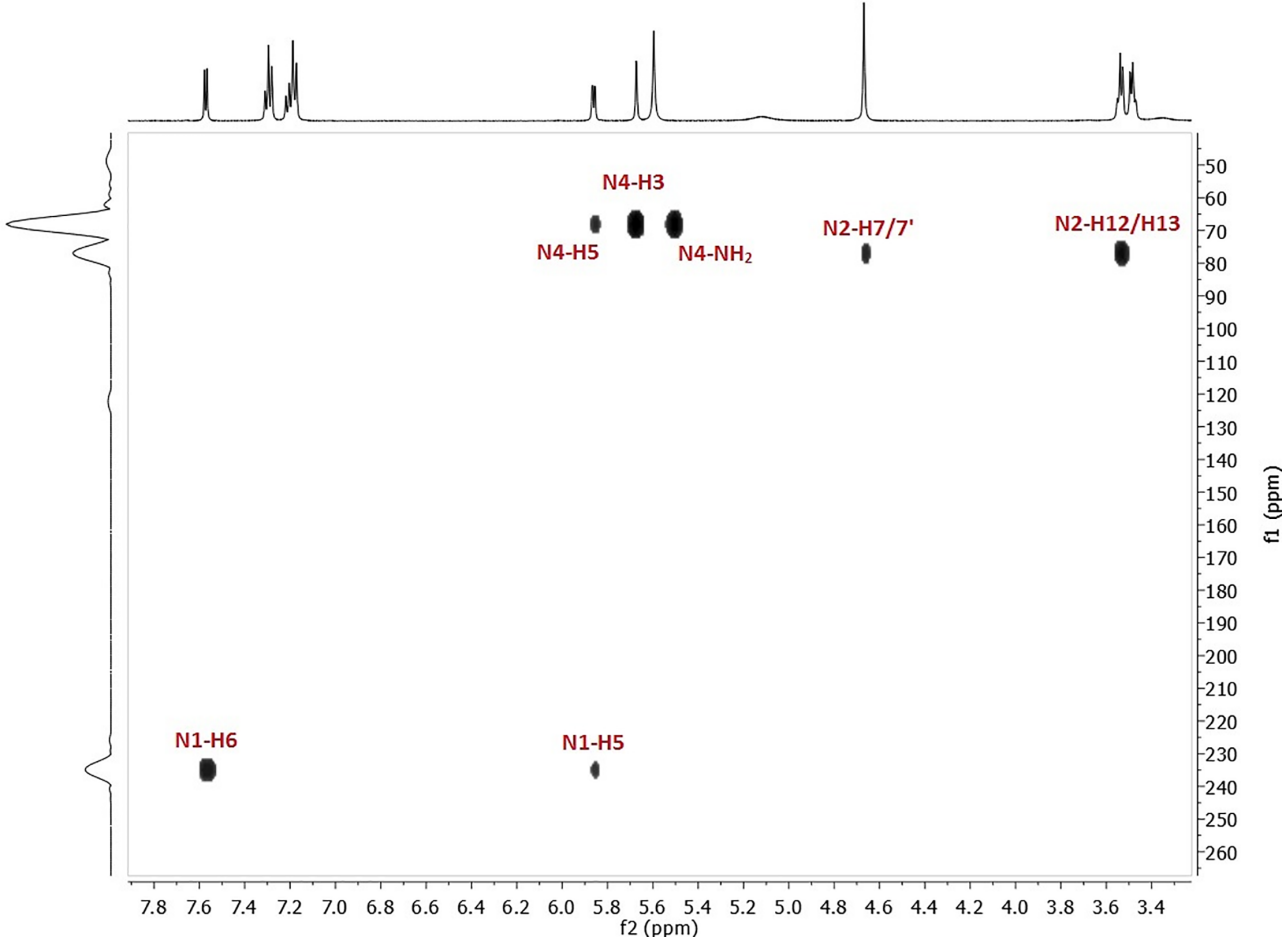
Correlation peaks between nitrogen and protons in the HMBC spectra of 1.

The assignment of the nitrogen signals was based on ^1^H-^15^N HSQC (S2 Table) and HMBC (Fig 2) spectra analysis. In the 2D HMBC spectrum (Fig 2), *N*1 resonance is observed at 234.38 ppm with cross-peaks to **H6** and **H5** protons. The position of the amino group on the pyridine ring is crucial and, based on the electronic effect, the 4-amino functionality influences the pyridinic nitrogen the most. The *N*2 signal is observed at 77.27 ppm with correlations to **H12**, **H13** and also to **H7/7′** protons. The *N*4 amino group signal is detected at 68.29 ppm with cross-peaks to **H6**, **H5**, and **H3** protons. In the HSQC spectrum, the *N*4 signal is observed at the same chemical shift value, but with a cross-peak only to the 4-NH_2_ signal in the ^1^H NMR spectrum. The assignment of the remaining protons and all carbon atoms of **1** is given in the supporting information (S1 and S2 Tables).

The proton affinity studies were performed by successive additions of 0.5, 1, 2 equiv of aq HCl to the same sample. After each addition, the sample was lyophilized and diluted in DMSO-*d*_*6*_. Each sample was examined by a set of 1D and 2D NMR experiments, and the differences in chemical shifts were analyzed. The signals of the potentially protonated compound were assigned using the same strategy as for reference compound **1** (S7–S9 Tables).

### Chemical shift perturbations

The NMR experiments reveal that the differences in chemical shifts between protons of reference compound **1** and its protonated analogs are negligible, apart from those for the protons in proximity of the protonation sites.

When the first portion of aq HCl was added (0.5 equiv), significant differences were observed in the resonances of **H5**, **H3** and the 4-amino group in 1D and 2D spectra (^1^H, ^13^C, COSY, HSQC, HMBC). The **H5** signal goes downfield from 5.87 ppm to 6.11 ppm (Δδ = 0.24 ppm), whereas the signal for **H3** –from 5.67 ppm to 5.80 ppm (Δδ = 0.13 ppm). Furthermore, changes in the signal shapes of the **H12** and **H13** protons of the ethylene chain can be observed in the ^1^H NMR spectrum. In reference structure **1**, these appear as two triplets; while in the spectrum after acid addition, they form a singlet at 3.59 ppm. The biggest difference is observed for the 4-amino group whose signal goes downfield by about 1.10 ppm.

**Fig 3:**
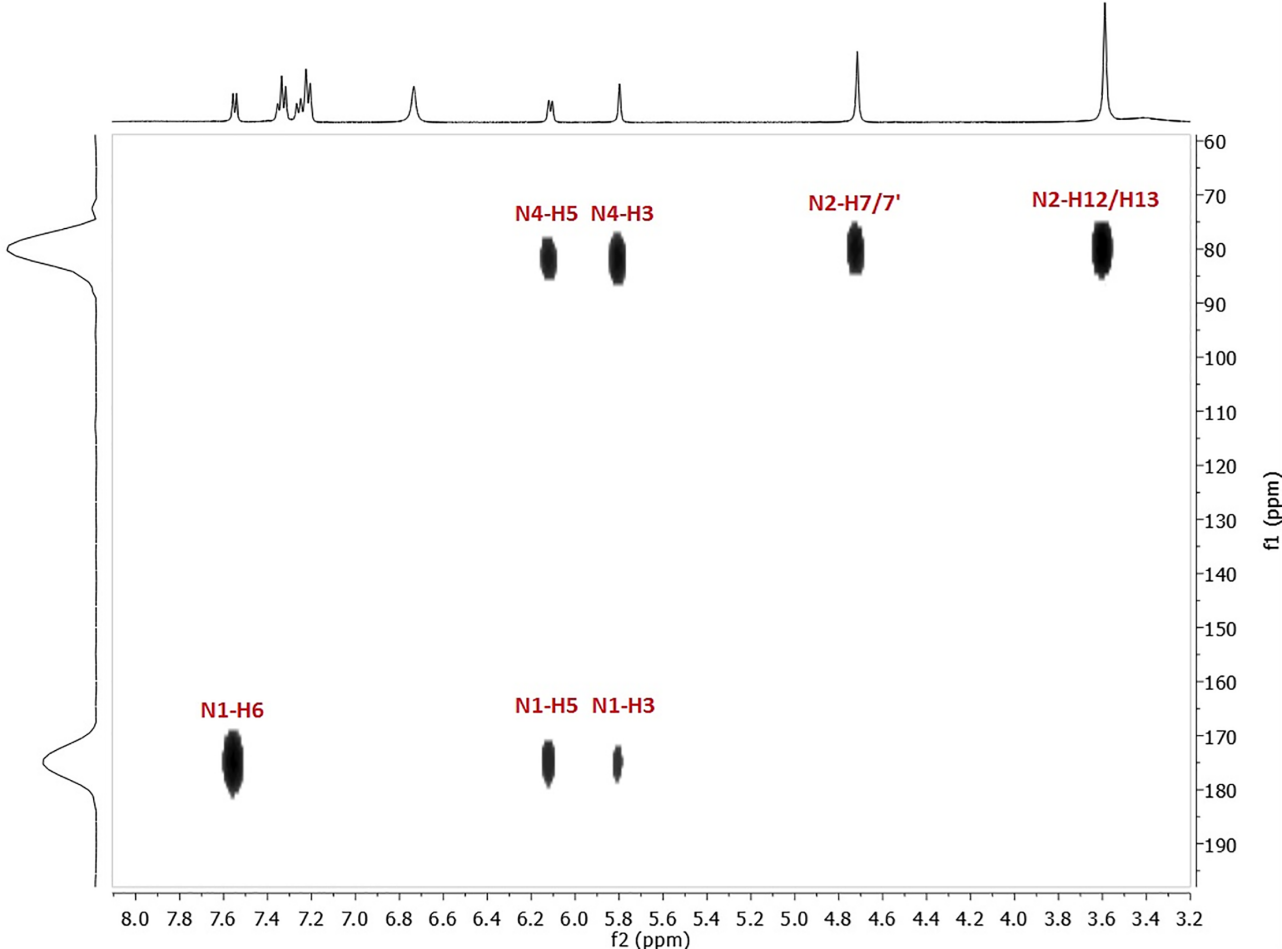
Correlation peaks between nitrogen and protons in the HMBC spectrum after addition of 0.5 equiv of HCl.

The HMBC plot shows that the N4 nitrogen signal is shifted downfield from 68.29 ppm to 81.52 ppm and is correlated to the 4-NH_2_ signal at 6.73 ppm (Fig 3). A significant change is also observed for the *N*1 nitrogen resonance that goes upfield to 175.51 ppm (Fig 3). In the ^13^C NMR spectrum, signal changes are detected as well. The most crucial changes are a C6 upfield shift from 147.61 ppm to 140.74 ppm (Δδ = 6.87 ppm) and a C4 shift from 156.05 ppm to 154.90 (Δδ = 1.15 ppm) (S3 Table)

**Fig 4:**
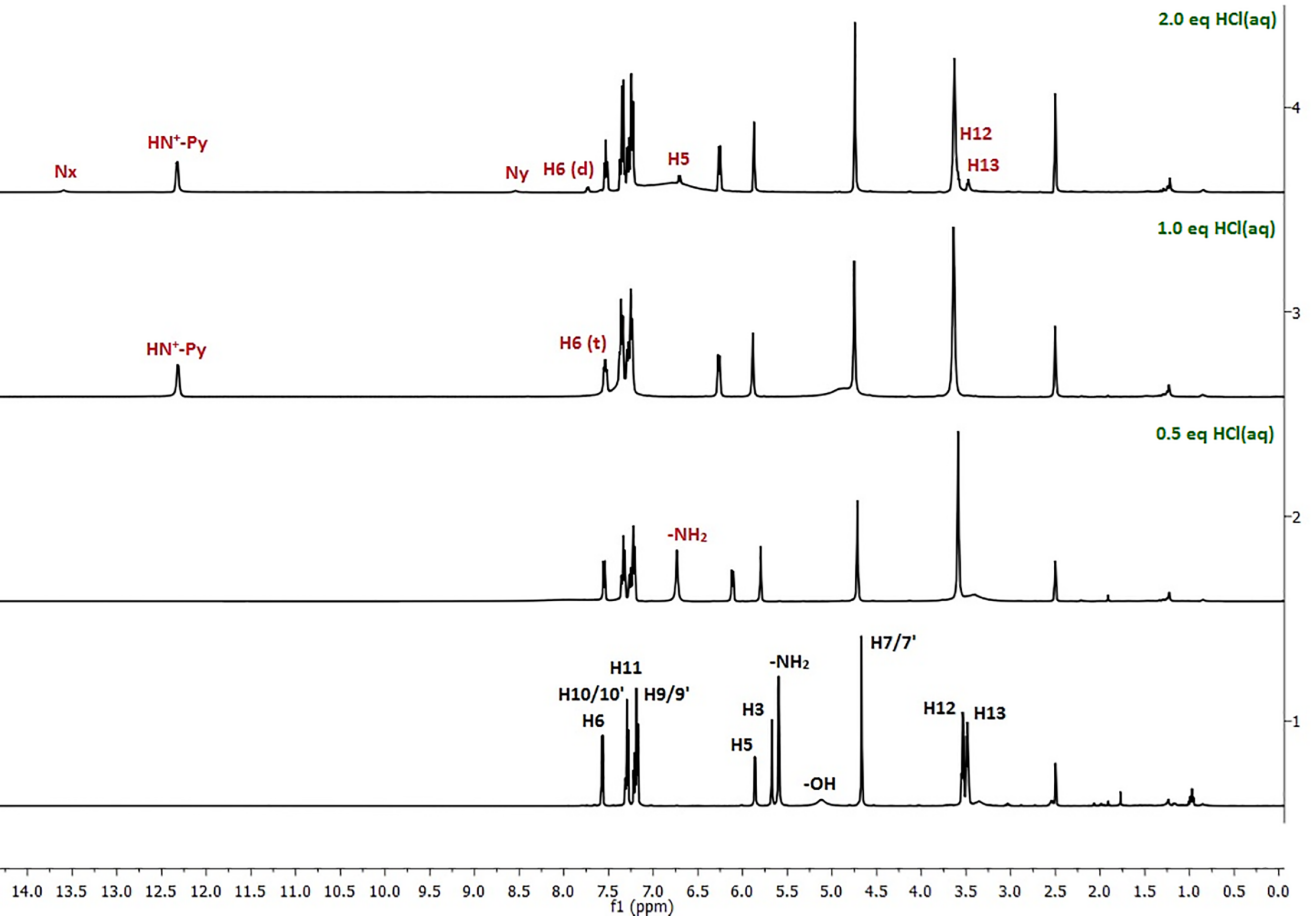
Comparison of the ^1^H NMR spectrum for unprotonated compound 1 (with proton assignment) and the spectra after addition of various equivs of HCl (only signals whose positions differ from the unprotonated compound are marked).

**Fig 5:**
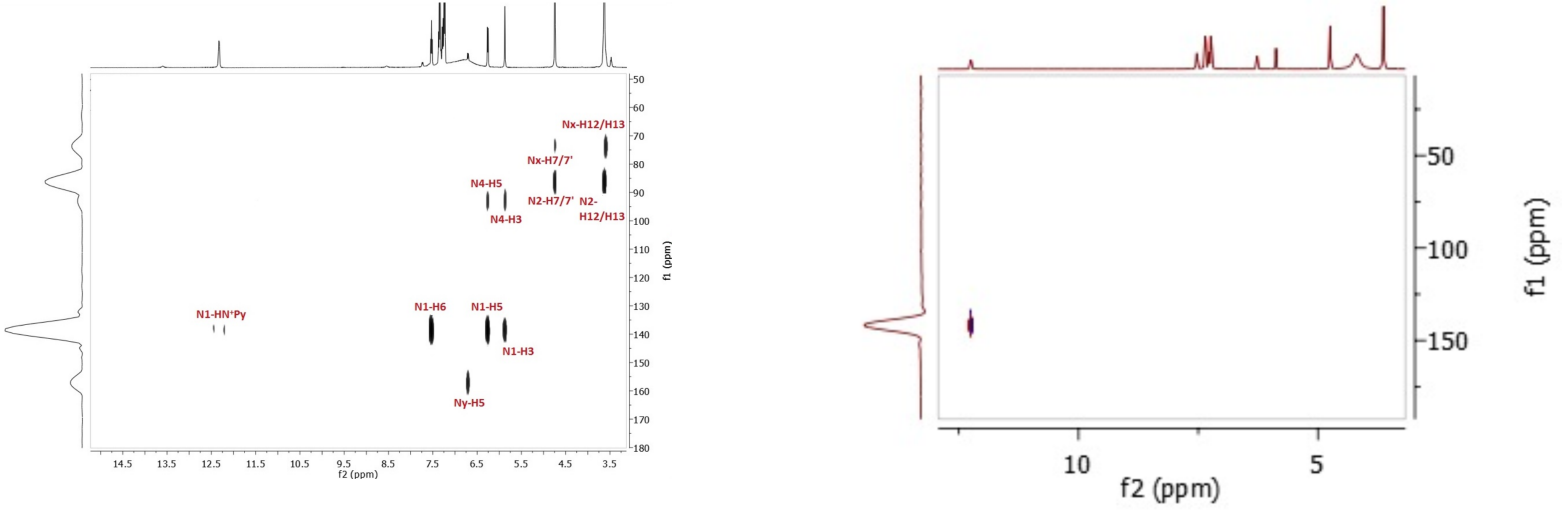
Correlation peaks between nitrogen and protons after addition of 1 equiv (HSQC spectrum, left side) and 2 equiv (HMBC spectrum, right side) of HCl.

The addition of another aq HCl portion results in downshifting of the **H5** resonance from 6.11 ppm to 6.26 ppm (Δδ = 0.15 ppm) in the ^1^H NMR spectrum (Fig 4). The signal of the 4-amino group is not observable–probably it moves downfield and overlaps with the phenyl proton multiplet. Moreover, a new signal at 12.25 ppm was visible in the spectrum which was assigned as an H attached to pyridine nitrogen N1. This protonation changed the multiplicity of **H6** changes from doublet to triplet (Fig 4) and this can be confirmed by the 1H-15N HSQC spectrum, which shows the N1 signal at 142.03 ppm with an observable cross-peak to H-N^+^Py (Fig 5). The proton attachment on *N*1 can be confirmed by the ^1^H-^15^N HSQC spectrum, which shows the *N*1 signal at 142.03 ppm with an observable cross-peak to H-N^+^Py (Fig 5). *N*2 resonance appears at 81.56 ppm and the *N*4 signal shifts downfield from 81.52 ppm to 90.01 ppm (Δδ = 8.49 ppm). Thus, the protonation increases shielding of the pyridine nitrogen and the signal appears at lower frequencies. The chemical shifts of carbons in the ^13^C NMR are not discussed herein, as their alterations are practically negligible (S5 Table).

Interesting changes are observed after the addition of 2 equiv of HCl. Some signals in each NMR spectrum (^1^H, ^15^N and ^13^C) are duplicated and have different chemical shifts, which could indicate that more than one protonated form is present in the mixture (Fig 4). In the ^1^H NMR spectrum, the **H6** signal is observed at 7.53 ppm as a triplet and an additional doublet at 7.73 ppm appears. The latter can be assigned to **H6** from another protonated structure. The same situation is observed for the duplicated **H5** signals that belong to two different protonated forms, with cross-peaks to the **H6** protons (Fig 4). In the ^1^H NMR spectrum, **H12** and **H13** in the predominant structure are still observed as one signal; however, two new resonances are detected at 3.47 ppm (t, *J* = 5.1 Hz) and 3.58 ppm (t, *J* = 5.6 Hz), again as separate triplets, as they are in the spectrum of **1**. Additionally, two new singlets of very low intensities at 13.60 ppm and 8.48 ppm from the second or even third co-structure appear in the spectrum. Consequently in the ^1^H-^15^N HSQC spectrum, cross-peaks between these new resonances, as well as correlated nitrogen atoms on ^15^N spectrum, are not visible. The ^1^H-^15^N HMBC spectrum (Fig 5) is slightly more informative then the HSQC one. The *N*1 signal appears at 141.85 ppm with a correlation signal to H-N^+^Py (δ = 12.25 ppm) in the HSQC spectrum and also to **H6** (δ = 7.73 ppm), H5 (δ = 6.26 ppm) and H3 (δ = 5.17 ppm) in the HMBC spectrum. The *N*2 nitrogen resonance is observed at 81.42 ppm with strong cross-peaks to **H7/7′** and **H12/13** protons, while the *N*4 signal is seen at 89.47 ppm, previously detected at 81.52 ppm (Fig 3), with cross-peaks to **H5** and **H3** protons from the major structure. Moreover, two new low intensity nitrogen signals are observed in the ^15^N spectra (Fig 5): an *N*x nitrogen at 67.40 ppm with cross peaks to **H7/7**′ and **H12/H13** protons from the minor structure, and an *N*y nitrogen at 163.39 ppm with a correlation to **H5** (δ = 6.71 ppm), attributable to the co-existing structure. In the ^13^C NMR spectrum, all carbon atoms signals are duplicated with mostly negligible shifts changes (S9 Table).

### Computational study

**Fig 6:**
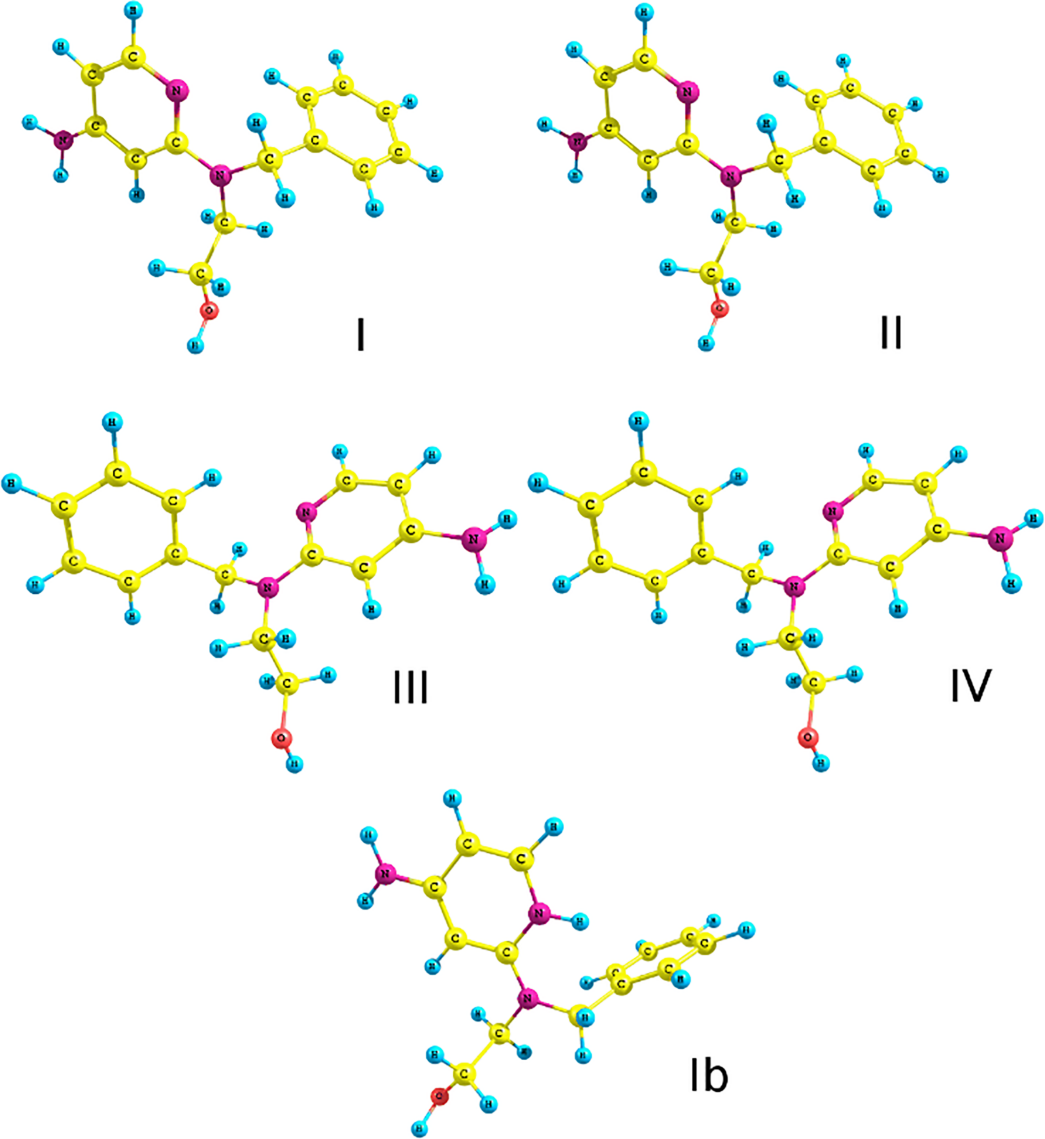
Conformations of pyridine derivatives (I–IV) and its tautomer (Ib) with the geometry of the most stable rotamers optimized at B3LYP/6-31G(d,p).

Given the possibility of multiple nitrogen atom protonation in **1**, we focused our attention during optimization on the four most stable rotamers, **I**–**IV**, and on tautomer **Ib**, all optimized at the B3LYP/6-31G (d, p) level of theory (Fig 6) [20, 21]. However, we did not take into account the quinoid tautomer **Ib** due to a significant total energy difference (single point calculations for optimized rotamer **I** and tautomer **Ib** at the B3LYP/6-311++G (2d,3p) level of theory) in 258.67 kcal/mol (0.412224 Hartree) in favor of the tautomer **I**. Analogously, we optimized the protonated form of **1** (rotamers **V**–**XVI**, S2–S4 Figs) rotamers **V**–**VIII** in S2 Fig for the *N1*-H, rotamers **IX**-**XII** in S3 Fig for the *N2*-H, and rotamers **XIII**-**XVI** in S4 Fig for *N*3-H given in SI). In order to determine the changes in the aromaticity of the pyridine ring after single nitrogen atom protonation, the HOMA [22] index was established for the following rotamers: **I** (neutral form), **V** *(N*1 protonated), **IX** (*N*2 protonated), and **XIII** (*N*4 protonated). The HOMA values for rotamers **V**, **IX** and **XII** were, respectively, 0.8049, 0.9309 and 0.8131 in comparison with the 0.9363 estimated for neutral rotamer **I**.

The protonation phenomenon was also investigated using the proton affinity (PA) descriptor in gaseous phase. The PA descriptor, calculated according to the equation: PA_H_ = H_ArN_ + H_H_^+^–H_ArNH_^+^, describes the susceptibility to heterolytic fission and thermodynamic stability of the N-H bond. The lower the PA value is, the more stable the compound [23, 24]. In the case of rotamers protonated at *N*1, *N*2 and *N*4 (**V**, **IX**, and **XIII**, S2–S4 Figs) in comparison with their corresponding deprotonated analogs (rotamers **XVII**–**XIX** given in the SI, optimized at the same level of theory as their corresponding protonated rotamers: **V**, **IX** and **XIII**), the PA parameter equals 244.86, 227.79, and 210.24 kcal/mol, respectively. Moreover, the most difficult proton elimination takes place for the exoamine group NH_3_^+^ (nitrogen atom *N*4, rotamer **XIII**). The obtained data show that the deprotonation does not have a significant influence on the geometry of compound **1**.

To get an insight into the protonation of the pyridinyl derivative, we simulated interactions between its neutral forms (rotamers **I**–**IV**) and HCl molecules. The optimization of the adducts of a neutral derivative with HCl was performed at the B3LYP/6-31G(d,p) level of theory, using very tight criteria inserted as a key-word in the input files [25]. From this experiment, the distance between the HCl molecule and *N*1, *N*2, *N*4 atoms of the most stable rotamer **I** (adducts **XX**, **XXIV** and **XXVIII**, S5 Fig) are 1.101, 2.719, and 1.798 Å, respectively, which suggests that the most favored contacts correspond to the following interactions: HCl–*N*1 and HCl–*N*4 (**XX** and **XXVIII** adducts). The optimization of adduct **XXIV** for protonation of *N*2 led to a structure with a protonated *N*1 atom instead of *N*2. This outcome was additionally verified by estimating the interaction energy between HCl and the pyridine derivative in the adducts (B3LYP/6-311++G(2d,3p) level of theory) involving the basis-set superposition error (BSSE) and counterpoise corrected method [26]. The results for adducts **XX**, **XXIV** and **XXVIII** are as follows: −60.09 (protonation of *N*1), −60.13 (protonation of *N*2) and −6.31 kcal/mol (protonation of *N*4). For **XXIV** adduct, regarding the interaction energy calculation and considering the BSSE factor, protonation in the presence of HCl still occurs on the *N*1 atom (S6 Fig).

Our next approach was the simulation of ^1^H NMR spectra (GIAO method) [27, 28] at the B3LYP-631G(d,p) level of theory, using TMS as a reference (S10–S16 Tables). The results of chemical shift calculations (ppm) show that the lower value of mean absolute deviation error (MAD) is obtained for rotamers *N*1- or *N*4-protonated (*N*-H^+^ and *N*-HCl type of adducts). For the *N*1-protonated molecules MAD is 0.81 (protonation *N*1–H^+^) or 0.96 (adduct *N*1–HCl, Table 1), while for the *N*4-protonated derivatives, MAD equals 0.81 (protonation *N*4–H^+^) or 0.73 (adduct *N*4–HCl, Table 2).

**Table 1:** Calculated and experimental data (at 293 K) for the optimized adducts XX–XXIII (adducts of I–IV with HCl in proximity of N1; experimental (δ_exp_) and calculated values of the chemical shifts (XX–XXIII), absolute errors (δ_XX_– δ_XXIII_), average absolute error (δ), relative percentage errors (Δδ); calculated NMR shielding for proton H_ref_ = 31.755 ppm for TMS (B3LYP/6-31G(d,p)/GIAO/gas); MAD = 0.96.

Locant	δ_exp_	XX	XXI	XXII	XXIII	δ_XX_	δ_XXI_	δ_XXII_	δ_XXIII_	Δ	Δδ
**H6**	7.58	8.08	8.05	8.05	8.05	0.50	0.47	0.47	0.47	0.48	**6**
**H5**	5.87	5.67	5.66	5.66	5.66	0.20	0.21	0.21	0.21	0.21	**4**
**H3**	5.67	5.29	5.22	5.22	5.22	0.38	0.45	0.45	0.45	0.43	**8**
**H9, H9'**	7.18	7.97	8.02	8.02	8.02	0.79	0.84	0.84	0.84	0.83	**12**
**H10, H10'**	7.3	7.39	7.42	7.42	7.42	0.09	0.12	0.12	0.12	0.11	**2**
**H11**	7.22	7.37	7.39	7.39	7.39	0.15	0.17	0.17	0.17	0.17	**2**
**NH2**	5.63	3.47	3.43	3.43	3.43	2.16	2.20	2.20	2.20	2.19	**39**
**OH**	5.12	0.03	0.05	0.05	0.05	5.09	5.07	5.07	5.07	5.08	**99**
**H7, H7'**	4.67	5.05	5.12	5.12	5.12	0.38	0.45	0.45	0.45	0.43	**9**
**H12**	3.49	3.24	3.25	3.25	3.25	0.25	0.24	0.24	0.24	0.24	**7**
**H13**	3.54	3.91	3.88	3.88	3.88	0.37	0.34	0.34	0.34	0.35	**10**

**Table 2:** Calculated and experimental data (at 293 K) for optimized rotamers XXVIII–XXXI (adducts of I–IV with HCl initially in proximity of N4); experimental (δ_exp_) and calculated values of the chemical shifts (XXVIII–XXXI), absolute errors (δ_XXVIII_– δ_XXXI_), average absolute error (δ), relative percentage errors (Δδ); calculated NMR shielding for proton H_ref_ = 31.755 ppm for TMS (B3LYP/6-31G(d,p)/GIAO/gas; MAD = 0.73.

Locant	δ_exp_	XXVIII	XXIX	XXX	XXXI	δ_XXVIII_	δ_XXIX_	δ_XXX_	δ_XXXI_	Δ	Δδ
**H6**	7.58	8.10	8.11	8.11	8.12	0.52	0.53	0.53	0.54	0.53	**7**
**H5**	5.87	5.89	5.92	5.89	5.95	0.02	0.05	0.02	0.08	0.05	**1**
**H3**	5.67	5.82	5.83	5.75	5.91	0.15	0.16	0.08	0.24	0.16	**3**
**H9, H9'**	7.18	7.55	7.56	7.51	7.54	0.37	0.38	0.33	0.36	0.36	**5**
**H10, H10'**	7.3	7.32	7.35	7.39	7.34	0.02	0.05	0.09	0.04	0.05	**1**
**H11**	7.22	7.28	7.30	7.33	7.30	0.06	0.08	0.11	0.08	0.08	**1**
**NH2**	5.63	4.72	4.74	4.71	4.76	0.91	0.89	0.92	0.87	0.90	**16**
**OH**	5.12	0.07	0.09	0.07	0.10	5.05	5.03	5.05	5.02	5.04	**98**
**H7, H7'**	4.67	4.88	4.81	4.86	4.80	0.21	0.14	0.19	0.13	0.17	**4**
**H12**	3.49	3.21	3.19	3.15	3.19	0.28	0.30	0.34	0.30	0.31	**9**
**H13**	3.54	3.95	3.86	3.90	3.91	0.41	0.32	0.36	0.37	0.36	**10**

*N*2-Protonation results in higher MAD values: 0.94 (protonation *N*2–H^+^) and 1.10 (adduct *N*2–HCl). The calculated ^1^H NMR spectrum of rotamer **I** (a neutral form) has a MAD value of 0.88. Additionally, the highest values for absolute percentage error were found for the alkyl chain protons, as well as for the labile hydroxyl and amine protons.

The results were subsequently verified using classical molecular dynamics and *Gromacs 2016*.*4* software [29]. DFT calculations revealed that multiple positions are attainable for the interactions between compound **1** and its chemical environment. An approximate insight into their relative importance was gained using a molecular dynamics simulation with a classical force field (GAFF) [30]. The model employed in our study was a single molecule of a neutral form of compound **1** solvated in a 10 nm cubic box of TIP3P water [31] as a source of protons. The resulting radial pair distance distribution functions (rdf) for the contacts are presented in S6 Fig. The radial distribution function with simulation time shows that contacts between water molecules and *N*1 and *N*4 occur faster than for *N*2 atoms (S6 Fig).

Next, we conducted Dynamic Reaction Coordinate calculations (DRC) [32] employing the PM7 method (*Mopac 2016*) [33] in which total energy is conserved by default so that, as the ‘reaction’ proceeds over time, energy is transferred between kinetic and potential forms (S7 and S8 Figs). The interaction between the oxonium ion (diluted HCl) and the *N*1 and *N*4 atoms of rotamer **I** was considered. The first simulation revealed that the proton and the *N*1 atom bonded almost immediately and 503 structures were generated (XYZ formats of the first and the last step are shown in S7 Fig), where oxonium ion oscillated close to the *N*1 atom. As a result of the second simulation, 1737 structures were obtained (XYZ formats of the first and the last step are given in S8 Fig) and the unchanged oxonium ion was moved close to the amine group (*N*4 atom). Moreover, in both cases, the ethyl hydroxy chain on the *N*2 nitrogen was oriented toward the *N*1 atom. This is an additional proof that the *N*1 atom is the protonation site.

The computations involving the hydroxy group in proximity to the *N*1 or *N*4 atoms required an additional optimization (*Gaussian G16 A*.*03* software) of tautomers **Ic** and **Id**. (S9 Fig). This procedure was performed using the same approach as for rotamer **I**, and total energies of these isomers were computed using single point calculation at the B3LYP/6-311++G (2d,3p) level of theory. The selected structures had the hydroxyl group directed toward the *N*1 or *N*4 atoms. The total energy differences of the two rotamers are 65.64 kcal/mol (0.1045976 Hartree), in favor of tautomer **Ic** which has the lowest energy. The energy differences for rotamer **I** and tautomer **Ic**, in terms of the total energy of molecules, are approximately 4.5 kcal/mol (0.0071692 Hartree). Subsequently, rotamer **I** and its tautomers **Ic** and **Id** were optimized at the B3LYP/6-31G(d,p) level of theory using the PM7 hamiltonian (*Mopac 2016* software). This procedure resulted in tautomers **Ie**–**Ig** (S10 Fig), respectively. Their final heats of formation (HOF) equal 5.09, 1.30, and 2.91 kcal/mol, respectively. The distances between the hydroxyl proton and *N*1 atom for these rotamers are 5.782, 2.118, and 4.251 Å, respectively, while the distances between the OH group and *N*2 atom equal 4.952, 6.172, and 6.240 Å, respectively. These results confirm that the possible interactions of hydroxyl protons and nitrogen atoms within the investigated structure determine its stability and final estimated semi-empirical HOF value.

## Discussion

In our earlier NMR experiments for determining the cyclization kinetics of 2-PyTPG carbonates conducted under acidic pH, we observed that more than one protonated species appeared in the spectra. To investigate what kind of structure could appear at low pH as well as to study the proton affinity differences between the *N*1, *N*2, *N*4 nitrogen atoms, we combined the NMR experiments and the computational data. In the NMR spectra, initial changes can be observed after addition of 0.5 equiv HCl. These resulted in alternation of the electron distribution in the pyridine ring and, consequently, in downfield shifts of the 4-amino protons. The changes in *N*1 (Fig 3) and *N*4 resonances are in agreement with the previous data on the protonation of aminopyridines that occurs preferably at *N*1 [34]. We presume that all changes in the NMR spectra are due to the partial protonation of *N*1 nitrogen, even without visible *N*1-H correlation to **H6**. Scalar coupling between **H6** and *N*1-H could be observed due to longer time attachment of the proton to the endocyclic nitrogen, but because of the very rapid proton exchange, we detected weighted average shifts of two co-existing forms, i.e. the half-protonated and reference ones. Moreover, the *N*1 nitrogen signal visible in the ^1^H-^15^N HMBC plot is shifted upfield which confirms the partial protonation.

Another portion of acid (1 equiv) directly confirmed the calculated proton affinity of *N*1. The main, unambiguous evidence is observed for the **H6** resonance that changes from a doublet to a triplet, due to coupling with both **H5** and *N*1-H protons. The protonation on *N*1 nitrogen is also confirmed by the computational data, where the HOMA aromaticity index for the neutral form (rotamer **I**), as well as for its corresponding form protonated at the *N*2 nitrogen (rotamer **IX**), is almost identical. Thus, we conclude that the protonation of the *N*2 nitrogen atom linked to the hydroxyethyl moiety has no influence on the HOMA aromaticity index of the pyridine ring. On the contrary, the protonation of *N*1 (rotamer **V**) and *N*4 (rotamer **XIII**) nitrogen atoms decreases the HOMA aromaticity index (0.8049 and 0.8131, respectively), and does not affect the stability of the protonated structure. On this account, we assume that the protonation of *N*1 and *N*4 is preferred over the *N*2 protonation, if the latter ever occurs under experimental conditions. Furthermore, the chemical shifts obtained *in silico* are closely correlated with the experimental data, with the best accordance for the *N*1- and *N*4-protonated derivatives (S1 Table). However, until this experiment, we have not observed additional sites of protonation; in the NMR spectra only one structure was present. These results also explain why changes in electron distribution that led to the decrease in aromaticity affect the sensitivity of 2-PyTPG and hinder thermocyclization under acidic conditions. These findings also provide conclusive support for the observation that the formation of the an oxazaphospholidine ring is not correlated with pH value (*N*2 is involved in the formation of the ring). The assessment of the effect of protonation on the aromaticity of the 2-pyridinyl derivative is confirmed by previously obtained crystallographic data [17].

These results encouraged us to create a superacidic environment to see if we were able to detect any other protonation sites and if there was a correlation between the computational and experimental data. Therefore, in the next step and under similar conditions, we added another 1 equiv of HCl (the overall amount of acid was 2 equiv) and this led to quite interesting results. Apart from the protonated main structure, other species, protonated on nitrogens other than *N*1, are visible. Using the 1D and 2D NMR spectra, we wanted to determine the structures of these new species but the results were not conclusive. Besides the *N*1, *N*2 and *N*4 resonances from the major structure protonated on the *N*1 nitrogen (Fig 4), another two characteristic signals appear in the spectra. However, although we do not have a definitive proof about which nitrogen was protonated and if there is any possibility to detect multi-protonated structures, we can draw some conclusions. In the ^1^H-^15^N HSQC plot, new signals assigned as **Hx** (δ = 13.60 ppm) and Hy (δ = 8.48 ppm) do not have a direct correlation to any nitrogen. On the other hand, from the *N*x and *N*y atoms we can observe HMBC correlations to the protons **H7/7′**, **H12/H13** and **H5**, respectively. Thus, we can conclude that *N*x presumably stands for *N*2 and *N*y for *N*1, both from different structures however, the assignment of the proper structure is unfeasible. Nevertheless, based on the previous data for the resonance with the most downfield shift, we suggest that Nx probably corresponds to the *N*1 protonation whereas *N*y to the *N*2 protonation. Moreover, due to a lack of unambiguous evidence, it is difficult to assume the existence of two different protonated structures or a multi-protonated structure in thermodynamic equilibrium. In particular, in the computational study that included BSSE factor, protonation on *N*2 does not occur.

Thereafter, we decided to conduct an experiment with more equivalents of hydrochloric acid to obtain a second structure in excess (3 and 5 equiv). To our dissatisfaction, the signals were not much more intense and assignment of proton resonances (Hx and Hy) and determination of the protonated structure was still impossible. However, experiments with 2 equiv of HCl performed at 700 MHz enabled the assignment of Hx/Nx and Hy/Ny as they show two additional signals in the ^1^H spectrum (Fig 4). These two resonances have strong cross-peaks to the *N*4 nitrogen (δ = 96.31 ppm) in the ^1^H-^15^N HSQC spectrum (Fig 5) as a consequence of electron density changes and the inhibition of the amino proton exchange that allows separate signals to be observed in the spectrum[35]. Even though the quinoid tautomer is not taken into account in the computational study, the observable changes in the NMR data cannot exclude such a possibility. Moreover, there is a huge difference (~1 ppm) between *N*4-**Ha** (δ = 8.6 ppm) and *N*4-**Hb** (δ = 7.6 ppm) chemical shifts. In view of this result, we assume that it could be either a quinoid form (**Ib**) or a structure with a partial double bond between C2 and *N*2 because only the hydroxy ethyl chain interaction (hydrogen bonding) or tautomerism could hamper the amino proton exchange. These results are in agreement with the theoretical NMR and DRC simulations which show that, within the resulting geometries of the optimized rotamers, the hydroxy group is in proximity of *N*1 and *N*4 and can interact with them, allowing optimization of tautomers **Ic** and **Id** (S8 Fig). Thus, the pyridine nitrogen atom can play a significant role in interactions with other acidic protons. Subsequently, the already optimized rotamer **I** and tautomers **Ic** and **Id** were further optimized using PM7 Hamiltonian. In the resulting structures **Ie**–**Ig**, (S10 Fig) we analyzed the final heat of formation. These results support that the possible interaction of hydroxyl protons and especially *N*1 atom within the investigated compound determine the stability of the structure and the final semi-empirical HOF value. The computational data include interactions of *N*4 and *N*1 with the hydroxy group from the ethylene chain however, after analyzing the 1D NOE experiments at 700 MHz, we do not observe any special interactions, indicating that hydrogen bonding occurs. Although we cannot completely exclude such a possibility because it might be too weak to detect under these experimental conditions.

In the following experiment, using hyperacidic conditions (10 equiv HCl), we attempted to determine if we were able to observe only the multi-protonated form of **1** in the NMR spectrum. Both ^1^H and ^13^C spectra clearly show the presence of one multi-protonated structure, i.e. the minor structure in the previous experiments. In the ^1^H-^15^N HSQC spectrum, there is an *N*1 resonance at 165.44 ppm correlated to the previously observed proton at 13.67 ppm, and this is another evidence for the multi-protonated compound that is consistent with the literature data [36]. Upon partial protonation, the *N*1 signal is observed at 175.51 ppm, in the *N*1 single-protonated structure the resonance of *N*1 is at 141.51 ppm, while in the multi-protonated form it is again shifted downfield to 163 ppm. Moreover, in the ^13^C NMR spectrum of mono-protonated structure, we can observe only the resonances previously assigned to the minor structure (S9 Table).

## Conclusion

From the above discussion, we conclude that protonation in the precursor is a key factor in the electron distribution and is strongly correlated with the thermolability of the 2-pyridinyl moiety. Based on a combined experimental and computational analysis, we have shown the preference for the protonation of the *N*1 atom with a simultaneous decrease in pyridine aromaticity. Structural and electronic features of protonated 2-pyridinyl may have important biological consequences for the chemical decomposition through thermocyclization. This corresponds with the previously reported zwitterionic form of [*N*-(pyridin-4-yl)]phosphoramidate for which anti-HIV activity was postulated [37]. The electron flow into the pyridine ring from the *N*2 atom and subsequent shortening of the bond between the C2 and *N*2 atoms was observed in an acidic environment. This observation correlates with already published crystallographic data [17], where the length of the C-*N*2 bond is much shorter than the typical single bonding.

Understanding the electron distribution allows control of the stability of the thermolabile 2-Pyridinyl group during the irreversible processes involving protection and subsequent removal of the protective group. The data presented herein logically imply the role of the *N*2 atom in cyclization under acidic conditions and its correlation with the reversible process concluded in the recovery of 2-pyridinyl thermolytic devices.

## Material and methods

### General methods and materials

All reagents and solvents (analytical grade, anhydrous) were obtained from commercial suppliers and used without further purification. All other compounds used were synthesized or commercially available and dried before use when necessary (P2O5 or lyophilization, molecular sieves 4Å). For TLC analysis, the precoated plates (Merck silica gel F254) were used, and for column chromatography silica gel Si 60, 35–70 mesh (Merck) was used. Mass spectra were recorded with ESI (electrospray ionization) technique, on mass spectrometer MicroTofQ Brucker Daltonic. All NMR spectra (1D and 2D) were carried out using Bruker Avance 400/500/700 MHz spectrometers. Spectral analysis was performed with Bruker's TOPSPIN/ MESTRANOVA simulation program. The 15N NMR spectra were measured in 5 mm sample tubes, at 20°C. Chemical shifts, on 15N predicted spectra, were determined using liquid NH3 as a primary reference.

### NMR analysis of 4-Amino-2-Pyridinyl-*N*-Benzylaminoethanol

Compound for NMR studies, 4-amino-2-pyridinyl-N-benzylaminoethanol (4-APyBzOH) was synthesized according to previously reported method [10]. 4-APyBzOH was diluted in DMSO-d6 (dried over 4Å molecular sieves). NMR measurement: 1H, 13C, 15N (COSY, HSQC and HMBC) of 4-APyBzOH were performed with the usage of aqueous HCl as a protonation agent. After each dosage of acid (0.5 eq, 1 eq 2 eq), measurement was performed two times–before and after water evaporation. If no differences have been seen in the spectra, then only experiments after evaporation were described (S1–S9 Tables). We have chosen DMSO-d6 as a solvent for few reasons: good solubility of compound before and after protonation, signals of–OH and amino groups are more likely to be seen on the spectra, which also influence the quality of two-dimensional correlation 1H-15N NMR and detection of nitrogen signals. Moreover, because of DMSO melting point at 19°C temperature of each experiment could not be lower than 20°C.We also considered to determine the solvent effect on 1H, 13C and 15N NMR shifts in our compound (non-polar solvent, hydrogen bonding solvent), however, this was not the principle of this study.

### Computational methods

Density functional calculations were executed, using the Gaussian 16 A.03 program, namely the B3LYP/6-31G(d,p), approach in the gaseous phase very tight criteria, keywords: Opt = vtight Int = ultrafine) [38] The vibrational frequencies and thermodynamic properties were calculated by applying the ideal gas, rigid rotor and harmonic oscillator approximations, and the energy minimum was confirmed by the frequency calculation for all rotamers; no negative frequencies were detected in the generated vibrational spectrum of the analyzed rotamers. All of were obtained by rotating the bonds Caromatic–Caliphatic, Caliphatic–Caliphatic, Naromatic–Caliphatic, Naliphatic–Caliphatic in torsion angle increments of 20°. The NMR shielding for proton (Href) was calculated for TMS (tetramethylsilane) at the B3LYP/6-31G(d,p) level of theory (gaseous phase) at 293 K. Particular clusters with hydrochloric acid molecules were constructed by adding HCl molecules to nitrogen atoms and then optimized. Experimental values of chemical shifts are given in ppm and are in good agreement with reference data; S1–S9 Tables). The 1H-NMR spectra were recorded in DMSO-d6. The compound of interest (1) and reference compound (TMS) were calculated using the same method, and the reference compound was used to obtain the chemical shifts of A according to the following equation: δi = σref − σi, where δi was the chemical shift of i-nuclei of A and σref and σi were the calculated isotropic magnetic shielding tensor for the TMS and A, respectively [39]. The calculated chemical shifts for the homotopic protons were averaged. The Chemcraft 1.7 software was utilized for visualization of all optimized rotamers [40]. The classical molecular dynamics calculations with the GROMACS 2016.4 suite [41] employed the GAFF general organic force field [42] and TIP3P water model [43]. The molecules were immersed in a 10 nm cubic box of water. To remove bad contacts, 1000 steps of minimization was carried out, then a two-stage equilibration was performed. First, an NVT (constant number, volume, and temperature conditions) run at 300 K was carried out for 0.3 ns, and then an NPT (constant number, pressure, and temperature conditions) run at 300 K and 1 atm was performed for 0.2 ns. Finally the NPT production run lasted for 5 ns. The timestep was 1 fs, and PME electrostatics with 10 Å real space nonbonded cutoff was employed. The trajectory was analysed using the VMD 1.9.1 program [44]. The semiempirical PM7 calculations were carried out using Mopac 2016 software [45].

## Supporting information

S1 Table ^1^H and ^13^C resonance assignment for reference structure for compound 1. (PDF)

S2 Table ^15^N resonance assignment for reference structure for compound 1. (PDF)

S3 Table ^1^H and ^13^C resonance assignment after 0.5 eq of aqueous HCl addition. (PDF)

S4 Table ^15^N resonance assignment after 0.5 eq of aqueous HCl addition. (PDF)

S5 Table ^1^H and ^13^C resonance assignment after 1 eq of aqueous HCl addition. (PDF)

S6 Table ^15^N resonance assignment after 1 eq of aqueous HCl addition. (PDF)

S7 Table ^1^H and ^13^C resonance assignment for major structure after 2eq of aqueous HCl addition. (PDF)

S8 Table ^15^N resonance assignment for major structure after 2eq of aqueous HCl addition. (PDF)

S9 Table ^1^H and ^13^C resonance assignment for co-existing structure after 2eq of aqueous HCl addition. (PDF)

S10 Table Calculated and experimental data of optimized neutral rotamers I–IV recorded at 293 K; experimental (δ_exp_) and calculated values of the chemical shifts (I–IV), absolute errors (δ_I_– δ_IV_), average absolute error (δ), relative percentage errors (Δδ); calculated NMR shielding for proton H_ref_ = 31.755 ppm for TMS (B3LYP/6-31G(d,p)/GIAO/gas; MAD = 0.88. (PDF)

S11 Table Calculated and experimental data of optimized protonated rotamers V–VIII recorded at 293 K; experimental (δ_exp_) and calculated values of the chemical shifts (V–VIII), absolute errors (δ_V_– δ_VIII_), average absolute error (δ), relative percentage errors (Δδ); calculated NMR shielding for proton H_ref_ = 31.755 ppm for TMS (B3LYP/6-31G(d,p)/GIAO/gas; MAD = 0.81. (PDF)

S12 Table Calculated and experimental data of optimized adducts XX–XXIII (adducts of I–IV with HCl located at N1) recorded at 293 K; experimental (δ_exp_) and calculated values of the chemical shifts (XX–XXIII), absolute errors (δ_XX_– δ_XXIII_), average absolute error (δ), relative percentage errors (Δδ); calculated NMR shielding for proton H_ref_ = 31.755 ppm for TMS (B3LYP/6-31G(d,p)/GIAO/gas; MAD = 0.96. (PDF)

S13 Table Calculated and experimental data of optimized protonated rotamers IX–XII recorded at 293 K; experimental (δ_exp_) and calculated values of the chemical shifts (IX–XII), absolute errors (δ_IX_– δ_XII_), average absolute error (δ), relative percentage errors (Δδ); calculated NMR shielding for proton H_ref_ = 31.755 ppm for TMS (B3LYP/6-31G(d,p)/GIAO/gas; MAD = 0.94. (PDF)

S14 Table Calculated and experimental data of optimized rotamers XXIV–XXVII (adducts of I–IV with HCl located at N2) recorded at 293 K; experimental (δ_exp_) and calculated values of the chemical shifts (XXIV–XXVII), absolute errors (δ_XXIV_– δ_XXVII_), average absolute error (δ), relative percentage errors (Δδ); calculated NMR shielding for proton H_ref_ = 31.755 ppm for TMS (B3LYP/6-31G(d,p)/GIAO/gas; MAD = 1.10. (PDF)

S15 Table Calculated and experimental data of optimized protonated rotamers XIII–XVI recorded at 293 K; experimental (δ_exp_) and calculated values of the chemical shifts (XIII–XVI), absolute errors (δ_XIII-_δ_XVI_), average absolute error (δ), relative percentage errors (Δδ); calculated NMR shielding for proton H_ref_ = 31.755 ppm for TMS (B3LYP/6-31G(d,p)/GIAO/gas; MAD = 0.85. (PDF)

S16 Table Calculated and experimental data of optimized rotamers XXVIII–XXXI (adducts of I–IV with HCl initially located at N4) recorded at 293 K; experimental (δ_exp_) and calculated values of the chemical shifts (XXVIII–XXXI), absolute errors (δ_XXVIII_– δ_XXXI_), average absolute error (δ), relative percentage errors (Δδ); calculated NMR shielding for proton H_ref_ = 31.755 ppm for TMS (B3LYP/6-31G(d,p)/GIAO/gas; MAD = 0.73. (PDF)

S17 Table Cartesian coordinates of analyzed rotamers. (PDF)

S1 Fig General mechanism of 2PyTPG thermocyclization based on the carbonates. (TIF)

S2 Fig Conformations of pyridine derivatives protonated in N1 with the geometry of most stable rotamers optimized at B3LYP/6-31G(d,p) (very tight criteria). (TIFF)

S3 Fig Conformations of pyridine derivatives protonated in N2 with the geometry of most stable rotamers optimized at B3LYP/6-31G(d,p) (very tight criteria). (TIFF)

S4 Fig Conformations of pyridine derivatives protonated in N4 with the geometry of most stable rotamers optimized at B3LYP/6-31G(d,p) (very tight criteria). (TIFF)

S5 Fig Conformation of pyridine derivatives–HCl adducts considering protonation of N1, N2 and N4 optimized at B3LYP/6-31G(d,p) (very tight criteria). (TIFF)

S6 Fig Radial distribution function of distances between the rotamer I and the water environment resulted from the classical molecular dynamics simulation (a–interaction of N1, b—interaction of N2, c—interaction of N4).
(TIFF)

S7 Fig SRC simulation considering interaction of H_3_O^+^ with N1 within the adduct I–H_3_O^+^ (XVIIa–first step, XVIIb–last step); XYZ coordinates of adducts I–H_3_O^+^ (XVIIIa–first step, XVIIIb–last step) onsidering interaction of H_3_O^+^ with N2 are given below.
(TIFF)

S8 Fig DRC simulation considering interaction of H_3_O^+^ with N4 within the adduct I–H_3_O^+^ (XIXa–first step, XIXb–last step). (TIFF)

S9 Fig Conformations of pyridine derivatives with the geometry of most rotamers (Ic and Id) optimized at B3LYP/6-31G(d,p) (very tight criteria). (TIFF)

S10 Fig Conformations of pyridine derivatives with the geometry rotamers optimized using PM7 method. (TIFF)
