# Supplementary material for: Experimental and computational studies on a protonated 2-pyridinyl moiety and its switchable effect for the design of thermolytic devices
Source: PLoS One. 2018 Sep 20;13(9):e0203604. doi: 10.1371/journal.pone.0203604 (PMC6147472; doi:10.1371/journal.pone.0203604)
Supplement: S1 Table — (PDF) [file pone.0203604.s001.pdf]

**Table S1.** Resonances and proton assignment for reference structure for compound **1.**

| | **H6** | **H5** | **H3** | **H7/7'** | **H9/9'** | **H10** | **H11** | **H12** | **H13** | **-NH2** | **-OH** |
|---|---|---|---|---|---|---|---|---|---|---|---|
| **σ($^1$H) [ppm]** | d, 7.58 | dd, 5.87 | d, 5.67 | s, 4.67 | m, 7.20-7.16 | t, 7.30 | t, 7.22 | t, 3.49 | t, 3.54 | s, 5.63 | s, 5.12 |
| ***J* [Hz]** | 5.6 | 5.6; 1.8 | 1.8 | - | - | 7.4 | 7.4 | 5.3 | 5.3 | - | - |
| | **C6** | **C5** | **C3** | **C7** | **C9** | **C10** | **C11** | **C12** | **C13** | **C4** | **C2** |
| **σ($^{13}$C) [ppm]** | 147.6 | 101.1 | 88.7 | 59.7 | 127.04 | 128.7 | 126.9 | 51.1 | 52.08 | 156.05 | 159.4 |

.
