# Supplementary material for: Experimental and computational studies on a protonated 2-pyridinyl moiety and its switchable effect for the design of thermolytic devices
Source: PLoS One. 2018 Sep 20;13(9):e0203604. doi: 10.1371/journal.pone.0203604 (PMC6147472; doi:10.1371/journal.pone.0203604)
Supplement: S2 Table — (PDF) [file pone.0203604.s002.pdf]

Table S2. Nitrogen assignment for compound **1**, based on ^{1}H-^{15}N HSQC and ^{1}H-^{15}N HMBC spectra.

	**HSQC**			**HMBC**		
	**N1**	**N2**	**N4**	**N1**	**N2**	**N4**
**σ(^{15}N) [ppm]**	-	-	68.29	234.38	77.27	68.29
