# Supplementary material for: Experimental and computational studies on a protonated 2-pyridinyl moiety and its switchable effect for the design of thermolytic devices
Source: PLoS One. 2018 Sep 20;13(9):e0203604. doi: 10.1371/journal.pone.0203604 (PMC6147472; doi:10.1371/journal.pone.0203604)
Supplement: S3 Table — (PDF) [file pone.0203604.s003.pdf]

Table S3. Resonance assignment after 0.5 eq of aqueous HCl addition and evaporation of water.

	**H6**	**H5**	**H3**	**H7/7’**	**H9/9’**	**H10/10’**	**H11**	**H12**	**H13**	**-NH2**	**-OH**	
**σ(^{1}H) [ppm]**	d, 7.55	dd, 6.11	d, 5.80	s, 4.72	7.22	t, 7.34	d, 7.26	s, 3.59	s, 3.59	s, 6.73	-	
***J* [Hz]**	6.5	6.5; 1.8	1.9	-	7.8	7.4	7.2	-	-	-	-	
	**C6**	**C5**	**C3**	**C7**	**C9**	**C10**	**C11**	**C12**	**C13**	**C4**	**C2**	**C8**
**σ(^{13}C) [ppm]**	140.7	102.2	88.3	59.2	126.9	129.03	127.4	51.8	52.8	154.9	158.2	137.9
