# Supplementary material for: Experimental and computational studies on a protonated 2-pyridinyl moiety and its switchable effect for the design of thermolytic devices
Source: PLoS One. 2018 Sep 20;13(9):e0203604. doi: 10.1371/journal.pone.0203604 (PMC6147472; doi:10.1371/journal.pone.0203604)
Supplement: S6 Table — (PDF) [file pone.0203604.s006.pdf]

Table S6. Nitrogen assignment after 1eq of aqueous HCl addition and water evaporation, based on ^{1}H-^{15}N HSQC and HMBC spectra.

	HSQC			HMBC		
	**N1**	**N2**	**N4**	**N1**	**N2**	**N4**
**σ(^{15}N) [ppm]**	142.03	-	-	142.03	81.56	90.01
