# Supplementary material for: Experimental and computational studies on a protonated 2-pyridinyl moiety and its switchable effect for the design of thermolytic devices
Source: PLoS One. 2018 Sep 20;13(9):e0203604. doi: 10.1371/journal.pone.0203604 (PMC6147472; doi:10.1371/journal.pone.0203604)
Supplement: S7 Table — (PDF) [file pone.0203604.s007.pdf]

Table S7. Proton assignment for major structure after 2eq of aqueous HCl addition and water evaporation.

	**H6**	**H5**	**H3**	**H7/7'**	**H9/9'**	**H10/10'**	**H11**	**H12**	**H13**	**H-N⁺Py**
**σ(^{1}H) [ppm]**	t, 7.53	dd, 6.26	s, 5.87	s, 4.74	d, 7.23	t, 7.34	t, 7.28	m, 3.69-3.59	m, 3.69-3.59	d, 12.25
***J* [Hz]**	6.6	7.1;1.8	-	-	7.8	7.4	7.3	-	-	6.2
	**C6**	**C5**	**C3**	**C7**	**C9**	**C10**	**C11**	**C12**	**C13**	**C4**
**σ(13C) [ppm]**	136.8	102.9	88.1	53.3	126.9	128.9	127.9	52.3	58.9	152.3
