# Supplementary material for: Experimental and computational studies on a protonated 2-pyridinyl moiety and its switchable effect for the design of thermolytic devices
Source: PLoS One. 2018 Sep 20;13(9):e0203604. doi: 10.1371/journal.pone.0203604 (PMC6147472; doi:10.1371/journal.pone.0203604)
Supplement: S8 Table — (PDF) [file pone.0203604.s008.pdf]

Table S8. Nitrogen assignment for major structure after 2eq of aqueous HCl addition and evaporation of water.

	HSQC			HMBC				
	**N1**	**N2**	**$N4_2$**	**N1**	**N2**	**N4**	**Nx**	**Ny**
**$\sigma(^{15}N)$ [ppm]**	141.85	-	-	141.85	81.42	89.47	67.40	163.39
