# Supplementary material for: Experimental and computational studies on a protonated 2-pyridinyl moiety and its switchable effect for the design of thermolytic devices
Source: PLoS One. 2018 Sep 20;13(9):e0203604. doi: 10.1371/journal.pone.0203604 (PMC6147472; doi:10.1371/journal.pone.0203604)
Supplement: S9 Table — (PDF) [file pone.0203604.s009.pdf]

Table S9. Proton assignment for co-existing minor structure after 2eq of aqueous HCl addition and water evaporation.

	**H6’**	**H5’**	**H3’**	**H7’’**	**H9’’**	**H10’’**	**H11’**	**H12’**	**H13’**	**H-N$^+$x**	**H-N$^+$y**
**σ(^{1}H) [ppm]**	d, 7.73	d, 6.71	s, 5.87	s, 4.74	d, 7.23	t, 7.34	t, 7.28	t, 3.47	t, 3.58	s, 13.60	s, 8.48
***J* [Hz]**	7.0	6.9	-	-	7.8	7.4	7.3	5.1	5.6	-	-
	**C6’**	**C5’**	**C3’**	**C7’**	**C9’**	**C10’**	**C11’**	**C12’**	**C13’**	**C4’**	**C2’**
**σ(13C) [ppm]**	137.3	105.4	104.0	54.1	128.2	129.2	127.7	53.9	58.7	152.5	157.5
