# Supplementary material for: Experimental and computational studies on a protonated 2-pyridinyl moiety and its switchable effect for the design of thermolytic devices
Source: PLoS One. 2018 Sep 20;13(9):e0203604. doi: 10.1371/journal.pone.0203604 (PMC6147472; doi:10.1371/journal.pone.0203604)
Supplement: S10 Table — (PDF) [file pone.0203604.s010.pdf]

S10 Table. Calculated and experimental data of optimized neutral rotamers I–IV recorded at 293 K; experimental (δ_{exp}) and calculated values of the chemical shifts (I–IV), absolute errors (δ_I– δ_{IV}), average absolute error (δ), relative percentage errors ($\Delta\delta$); calculated NMR shielding for proton H_{ref} = 31.755 ppm for TMS (B3LYP/6-31G(d,p)/GIAO/gas; MAD = 0.88.

Locant	δ_{exp}	I	II	III	IV	δ_I	δ_{II}	δ_{III}	δ_{IV}	Δ	Δδ
**H6**	7.58	7.89	7.90	7.89	7.90	0.31	0.32	0.31	0.32	0.31	**4**
**H5**	5.87	5.65	5.65	5.65	5.65	0.22	0.22	0.22	0.22	0.22	**4**
**H3**	5.67	5.50	5.49	5.50	5.50	0.17	0.18	0.17	0.17	0.17	**3**
**H9, H9'**	7.18	7.59	7.59	7.59	7.59	0.41	0.41	0.41	0.41	0.41	**6**
**H10, H10'**	7.3	7.31	7.31	7.31	7.31	0.01	0.01	0.01	0.01	0.01	**0**
**H11**	7.22	7.26	7.26	7.26	7.26	0.04	0.04	0.04	0.04	0.04	**1**
**NH2**	5.63	2.86	2.86	2.86	2.86	2.77	2.77	2.77	2.77	2.77	**49**
**OH**	5.12	0.14	0.14	0.14	0.14	4.98	4.98	4.98	4.98	4.98	**97**
**H7, H7'**	4.67	4.79	4.79	4.79	4.79	0.12	0.12	0.12	0.12	0.12	**3**
**H12**	3.49	3.18	3.18	3.18	3.18	0.31	0.31	0.31	0.31	0.31	**9**
**H13**	3.54	3.91	3.91	3.91	3.91	0.37	0.37	0.37	0.37	0.37	**11**
