# Supplementary material for: Experimental and computational studies on a protonated 2-pyridinyl moiety and its switchable effect for the design of thermolytic devices
Source: PLoS One. 2018 Sep 20;13(9):e0203604. doi: 10.1371/journal.pone.0203604 (PMC6147472; doi:10.1371/journal.pone.0203604)
Supplement: S11 Table — (PDF) [file pone.0203604.s011.pdf]

S11 Table. Calculated and experimental data of optimized protonated rotamers V–VIII recorded at 293 K; experimental (δ_{exp}) and calculated values of the chemical shifts (V–VIII), absolute errors (δ_V– δ_{VIII}), average absolute error (δ), relative percentage errors (Δδ); calculated NMR shielding for proton H_{ref} = 31.755 ppm for TMS (B3LYP/6-31G(d,p)/GIAO/gas; MAD = 0.81.

Locant	δ_{exp}	V	VI	VII	VIII	δ_V	δ_{VI}	δ_{VII}	δ_{VIII}	Δ	Δδ
**H6**	7.58	6.87	6.87	6.87	6.87	0.71	0.71	0.71	0.71	0.71	**9**
**H5**	5.87	5.93	5.93	5.93	5.93	0.06	0.06	0.06	0.06	0.06	**1**
**H3**	5.67	5.72	5.72	5.72	5.72	0.05	0.05	0.05	0.05	0.05	**1**
**H9, H9'**	7.18	7.50	7.51	7.50	7.50	0.32	0.32	0.32	0.32	0.32	**5**
**H10, H10'**	7.3	7.83	7.83	7.83	7.83	0.53	0.53	0.53	0.53	0.53	**7**
**H11**	7.22	7.87	7.87	7.87	7.87	0.65	0.65	0.65	0.65	0.65	**9**
**NH2**	5.63	4.46	4.46	4.46	4.46	1.17	1.18	1.17	1.17	1.17	**21**
**OH**	5.12	0.70	0.70	0.70	0.70	4.42	4.42	4.42	4.42	4.42	**86**
**H7, H7'**	4.67	4.45	4.45	4.45	4.45	0.22	0.22	0.22	0.22	0.22	**5**
**H12**	3.49	3.78	3.78	3.78	3.78	0.29	0.29	0.29	0.29	0.29	**8**
**H13**	3.54	4.06	4.06	4.06	4.06	0.52	0.52	0.52	0.52	0.52	**15**
