# Supplementary material for: Experimental and computational studies on a protonated 2-pyridinyl moiety and its switchable effect for the design of thermolytic devices
Source: PLoS One. 2018 Sep 20;13(9):e0203604. doi: 10.1371/journal.pone.0203604 (PMC6147472; doi:10.1371/journal.pone.0203604)
Supplement: S12 Table — (PDF) [file pone.0203604.s012.pdf]

S12 Table. Calculated and experimental data of optimized adducts XX–XXIII (adducts of I–IV with HCl located at N1) recorded at 293 K; experimental (δ_{exp}) and calculated values of the chemical shifts (XX–XXIII), absolute errors (δ_{XX}– δ_{XXIII}), average absolute error (δ), relative percentage errors (Δδ); calculated NMR shielding for proton H_{ref} = 31.755 ppm for TMS (B3LYP/6-31G(d,p)/GIAO/gas; MAD = 0.96.

Locant	δ_{exp}	XX	XXI	XXII	XXIII	δ_{XX}	δ_{XXI}	δ_{XXII}	δ_{XXIII}	Δ	Δδ
**H6**	7.58	8.08	8.05	8.05	8.05	0.50	0.47	0.47	0.47	0.48	**6**
**H5**	5.87	5.67	5.66	5.66	5.66	0.20	0.21	0.21	0.21	0.21	**4**
**H3**	5.67	5.29	5.22	5.22	5.22	0.38	0.45	0.45	0.45	0.43	**8**
**H9, H9'**	7.18	7.97	8.02	8.02	8.02	0.79	0.84	0.84	0.84	0.83	**12**
**H10, H10'**	7.3	7.39	7.42	7.42	7.42	0.09	0.12	0.12	0.12	0.11	**2**
**H11**	7.22	7.37	7.39	7.39	7.39	0.15	0.17	0.17	0.17	0.17	**2**
**NH2**	5.63	3.47	3.43	3.43	3.43	2.16	2.20	2.20	2.20	2.19	**39**
**OH**	5.12	0.03	0.05	0.05	0.05	5.09	5.07	5.07	5.07	5.08	**99**
**H7, H7'**	4.67	5.05	5.12	5.12	5.12	0.38	0.45	0.45	0.45	0.43	**9**
**H12**	3.49	3.24	3.25	3.25	3.25	0.25	0.24	0.24	0.24	0.24	**7**
**H13**	3.54	3.91	3.88	3.88	3.88	0.37	0.34	0.34	0.34	0.35	**10**
