# Supplementary material for: Experimental and computational studies on a protonated 2-pyridinyl moiety and its switchable effect for the design of thermolytic devices
Source: PLoS One. 2018 Sep 20;13(9):e0203604. doi: 10.1371/journal.pone.0203604 (PMC6147472; doi:10.1371/journal.pone.0203604)
Supplement: S13 Table — (PDF) [file pone.0203604.s013.pdf]

S13 Table. Calculated and experimental data of optimized protonated rotamers IX–XII recorded at 293 K; experimental (δ_{exp}) and calculated values of the chemical shifts (IX–XII), absolute errors (δ_{IX}– δ_{XII}), average absolute error (δ), relative percentage errors (Δδ); calculated NMR shielding for proton H_{ref} = 31.755 ppm for TMS (B3LYP/6-31G(d,p)/GIAO/gas; MAD = 0.94.

Locant	δ_{exp}	IX	X	XI	XII	δ_{IX}	δ_{X}	δ_{XI}	δ_{XII}	Δ	Δδ
**H6**	7.58	8.21	8.30	8.24	8.25	0.63	0.72	0.66	0.67	0.67	**9**
**H5**	5.87	6.57	6.69	6.62	6.61	0.70	0.82	0.75	0.74	0.75	**13**
**H3**	5.67	5.83	5.90	5.99	5.99	0.16	0.23	0.32	0.32	0.26	**5**
**H9, H9'**	7.18	7.46	7.63	7.64	7.60	0.28	0.45	0.46	0.42	0.40	**6**
**H10, H10'**	7.3	7.84	7.92	7.97	8.02	0.54	0.62	0.67	0.72	0.64	**9**
**H11**	7.22	7.95	8.00	8.04	8.13	0.73	0.78	0.82	0.91	0.81	**11**
**NH2**	5.63	4.16	4.32	4.28	4.26	1.47	1.31	1.35	1.37	1.38	**24**
**OH**	5.12	0.55	0.70	0.73	0.74	4.57	4.42	4.39	4.38	4.44	**87**
**H7, H7'**	4.67	4.50	4.59	4.67	4.59	0.17	0.08	0.00	0.08	0.08	**2**
**H12**	3.49	3.20	3.29	3.31	3.33	0.29	0.20	0.18	0.16	0.21	**6**
**H13**	3.54	4.20	4.34	4.32	4.33	0.66	0.80	0.78	0.79	0.76	**21**
