# Supplementary material for: Experimental and computational studies on a protonated 2-pyridinyl moiety and its switchable effect for the design of thermolytic devices
Source: PLoS One. 2018 Sep 20;13(9):e0203604. doi: 10.1371/journal.pone.0203604 (PMC6147472; doi:10.1371/journal.pone.0203604)
Supplement: S14 Table — (PDF) [file pone.0203604.s014.pdf]

S14 Table. Calculated and experimental data of optimized rotamers XXIV–XXVII (adducts of I–IV with HCl located at N2) recorded at 293 K; experimental (δ_{exp}) and calculated values of the chemical shifts (XXIV–XXVII), absolute errors (δ_{XXIV}– δ_{XXVII}), average absolute error (δ), relative percentage errors (Δδ); calculated NMR shielding for proton H_{ref} = 31.755 ppm for TMS (B3LYP/6-31G(d,p)/GIAO/gas; MAD = 1.10.

Locant	δ_{exp}	XXIV	XXV	XXVI	XXVII	δ_{XXIV}	δ_{XXV}	δ_{XXVI}	δ_{XXVII}	Δ	Δδ
**H6**	7.58	7.76	7.73	7.72	7.69	0.18	0.15	0.14	0.11	0.15	**2**
**H5**	5.87	4.92	4.86	4.92	4.95	0.95	1.01	0.95	0.92	0.96	**16**
**H3**	5.67	4.94	4.98	4.94	4.93	0.73	0.69	0.73	0.74	0.72	**13**
**H9, H9'**	7.18	7.53	7.58	7.54	7.40	0.35	0.40	0.36	0.22	0.33	**5**
**H10, H10'**	7.3	7.36	7.29	7.34	7.46	0.06	0.01	0.04	0.16	0.07	**1**
**H11**	7.22	7.26	7.17	7.22	7.46	0.04	0.05	0.00	0.24	0.08	**1**
**NH2**	5.63	3.11	3.12	3.10	3.10	2.52	2.51	2.53	2.53	2.52	**45**
**OH**	5.12	0.33	0.31	0.31	0.33	4.79	4.81	4.81	4.79	4.80	**94**
**H7, H7'**	4.67	3.46	3.51	3.50	3.40	1.21	1.16	1.17	1.27	1.20	**26**
**H12**	3.49	2.85	2.82	2.85	2.97	0.64	0.67	0.64	0.52	0.62	**18**
**H13**	3.54	4.1716	4.1148	4.18	4.15	0.63	0.57	0.64	0.61	0.61	**17**
