# Supplementary material for: Experimental and computational studies on a protonated 2-pyridinyl moiety and its switchable effect for the design of thermolytic devices
Source: PLoS One. 2018 Sep 20;13(9):e0203604. doi: 10.1371/journal.pone.0203604 (PMC6147472; doi:10.1371/journal.pone.0203604)
Supplement: S15 Table — (PDF) [file pone.0203604.s015.pdf]

S15 Table. Calculated and experimental data of optimized protonated rotamers XIII–XVI recorded at 293 K; experimental (δ_{exp}) and calculated values of the chemical shifts (XIII–XVI), absolute errors (δ_{XIII}-δ_{XVI}), average absolute error (δ), relative percentage errors (Δδ); calculated NMR shielding for proton H_{ref} = 31.755 ppm for TMS (B3LYP/6-31G(d,p)/GIAO/gas; MAD = 0.85.

Locant	δ_{exp}	XIII	XIV	XV	XVI	δ_{XIII}	δ_{XIV}	δ_{XV}	δ_{XVI}	Δ	Δδ
**H6**	7.58	8.78	8.78	8.78	8.78	1.20	1.20	1.20	1.20	1.20	**16**
**H5**	5.87	6.20	6.20	6.20	6.20	0.33	0.33	0.33	0.33	0.33	**6**
**H3**	5.67	5.89	5.89	5.89	5.89	0.22	0.22	0.22	0.22	0.22	**4**
**H9, H9'**	7.18	7.42	7.42	7.42	7.42	0.24	0.24	0.24	0.24	0.24	**3**
**H10, H10'**	7.3	7.67	7.67	7.67	7.67	0.37	0.37	0.37	0.37	0.37	**5**
**H11**	7.22	7.66	7.66	7.66	7.66	0.44	0.44	0.44	0.44	0.44	**6**
**NH2**	5.63	5.23	5.23	5.23	5.23	0.40	0.40	0.40	0.40	0.40	**7**
**OH**	5.12	0.44	0.44	0.44	0.44	4.68	4.68	4.68	4.68	4.68	**91**
**H7, H7'**	4.67	4.51	4.51	4.51	4.51	0.16	0.16	0.16	0.16	0.16	**3**
**H12**	3.49	4.25	4.25	4.25	4.26	0.76	0.76	0.76	0.76	0.76	**22**
**H13**	3.54	4.03	4.03	4.03	4.03	0.49	0.49	0.49	0.49	0.49	**14**
