# Supplementary material for: Experimental and computational studies on a protonated 2-pyridinyl moiety and its switchable effect for the design of thermolytic devices
Source: PLoS One. 2018 Sep 20;13(9):e0203604. doi: 10.1371/journal.pone.0203604 (PMC6147472; doi:10.1371/journal.pone.0203604)
Supplement: S16 Table — (PDF) [file pone.0203604.s016.pdf]

S16 Table. Calculated and experimental data of optimized rotamers XXVIII–XXXI (adducts of I–IV with HCl initially located at N4) recorded at 293 K; experimental (δ_{exp}) and calculated values of the chemical shifts (XXVIII–XXXI), absolute errors (δ_{XXVIII}–δ_{XXXI}), average absolute error (δ), relative percentage errors (Δδ); calculated NMR shielding for proton H_{ref} = 31.755 ppm for TMS (B3LYP/6-31G(d,p)/GIAO/gas; MAD = 0.73.

Locant	δ_{exp}	XXVIII	XXIX	XXX	XXXI	δ_{XXVIII}	δ_{XXIX}	δ_{XXX}	δ_{XXXI}	Δ	Δδ
**H6**	7.58	8.10	8.11	8.11	8.12	0.52	0.53	0.53	0.54	0.53	**7**
**H5**	5.87	5.89	5.92	5.89	5.95	0.02	0.05	0.02	0.08	0.05	**1**
**H3**	5.67	5.82	5.83	5.75	5.91	0.15	0.16	0.08	0.24	0.16	**3**
**H9, H9'**	7.18	7.55	7.56	7.51	7.54	0.37	0.38	0.33	0.36	0.36	**5**
**H10, H10'**	7.3	7.32	7.35	7.39	7.34	0.02	0.05	0.09	0.04	0.05	**1**
**H11**	7.22	7.28	7.30	7.33	7.30	0.06	0.08	0.11	0.08	0.08	**1**
**NH2**	5.63	4.72	4.74	4.71	4.76	0.91	0.89	0.92	0.87	0.90	**16**
**OH**	5.12	0.07	0.09	0.07	0.10	5.05	5.03	5.05	5.02	5.04	**98**
**H7, H7'**	4.67	4.88	4.81	4.86	4.80	0.21	0.14	0.19	0.13	0.17	**4**
**H12**	3.49	3.21	3.19	3.15	3.19	0.28	0.30	0.34	0.30	0.31	**9**
**H13**	3.54	3.95	3.86	3.90	3.91	0.41	0.32	0.36	0.37	0.36	**10**
