# Supplementary material for: Experimental and computational studies on a protonated 2-pyridinyl moiety and its switchable effect for the design of thermolytic devices
Source: PLoS One. 2018 Sep 20;13(9):e0203604. doi: 10.1371/journal.pone.0203604 (PMC6147472; doi:10.1371/journal.pone.0203604)
Supplement: S17 Table — (PDF) [file pone.0203604.s017.pdf]

S17 Table. Cartesian coordinates of analyzed rotamers (adducts)

Rotamer I

0 1

C	3.386127000	-2.002026000	0.206338000
C	2.220036000	-2.265955000	0.905428000
N	1.164983000	-1.447234000	0.979609000
C	1.243913000	-0.265234000	0.332858000
C	2.393148000	0.112240000	-0.395452000
C	3.475555000	-0.769238000	-0.470977000
N	0.138468000	0.562907000	0.424020000
C	-1.008830000	0.202095000	1.262939000
C	0.034133000	1.797853000	-0.334012000
N	4.593671000	-0.457917000	-1.226598000
C	-2.210800000	-0.297970000	0.479908000
C	-2.132671000	-1.491505000	-0.253797000
C	-3.234491000	-1.954462000	-0.969785000
C	-4.432526000	-1.233568000	-0.965462000
C	-4.521012000	-0.047760000	-0.238329000
C	-3.414250000	0.415067000	0.478523000
C	0.632981000	3.003911000	0.395834000
O	0.454073000	4.132275000	-0.451040000
H	4.198177000	-2.721303000	0.177755000
H	2.118974000	-3.207230000	1.444444000
H	2.458205000	1.072214000	-0.893377000
H	-0.666056000	-0.566543000	1.956137000
H	-1.300736000	1.082688000	1.849091000
H	0.512751000	1.692772000	-1.312487000
H	-1.023665000	1.996301000	-0.532099000
H	4.736727000	0.519040000	-1.437903000
H	5.445248000	-0.942682000	-0.984004000
H	-1.203170000	-2.052934000	-0.247366000
H	-3.161906000	-2.882179000	-1.530705000
H	-5.290003000	-1.596554000	-1.524911000
H	-5.447436000	0.519665000	-0.229009000
H	-3.488734000	1.339228000	1.047604000
H	0.122967000	3.134833000	1.363223000
H	1.696449000	2.818529000	0.610642000
H	0.791899000	4.910975000	0.007972000

Rotamer Ib

1 1

C	16.624110000	-25.340922000	-1.562465000
C	16.711435000	-26.692465000	-1.652756000
N	17.871688000	-27.353362000	-1.365564000
C	19.012910000	-26.703421000	-0.956062000
C	18.958738000	-25.306119000	-0.850724000
C	17.778491000	-24.605712000	-1.141337000
N	20.107133000	-27.459292000	-0.683562000

C	20.171115000	-28.880421000	-1.083015000
C	21.332708000	-26.839337000	-0.164625000
N	17.717725000	-23.263261000	-1.045356000
C	19.228139000	-29.809695000	-0.330713000
C	19.072266000	-29.720234000	1.060011000
C	18.238389000	-30.611699000	1.732007000
C	17.550026000	-31.601931000	1.024897000
C	17.696957000	-31.696841000	-0.358218000
C	18.531216000	-30.802252000	-1.034437000
C	22.274738000	-26.317385000	-1.255987000
O	23.304461000	-25.640583000	-0.565755000
H	15.695762000	-24.837513000	-1.802447000
H	15.879431000	-27.318553000	-1.950254000
H	19.844619000	-24.756631000	-0.566087000
H	19.992062000	-28.966146000	-2.162706000
H	21.206378000	-29.190609000	-0.920325000
H	21.075030000	-26.020943000	0.511479000
H	21.857205000	-27.582508000	0.440046000
H	18.510328000	-22.720771000	-0.739690000
H	16.870996000	-22.761818000	-1.261835000
H	19.603331000	-28.953614000	1.617218000
H	18.127579000	-30.537465000	2.809232000
H	16.903414000	-32.295790000	1.552384000
H	17.168209000	-32.465329000	-0.913052000
H	18.655752000	-30.891045000	-2.111640000
H	22.653339000	-27.160466000	-1.853725000
H	21.724156000	-25.653186000	-1.941603000
H	24.019699000	-25.435082000	-1.180978000
H	17.851086000	-28.368399000	-1.320756000

Rotamer Ic

0 1

C	16.781036000	-24.646955000	-1.599579000
C	18.071810000	-24.339632000	-1.211826000
N	19.042295000	-25.241240000	-1.001642000
C	18.732342000	-26.546831000	-1.157427000
C	17.438049000	-26.966798000	-1.524507000
C	16.448313000	-26.008855000	-1.764113000
N	19.749975000	-27.452867000	-0.928359000
C	19.676857000	-28.811912000	-1.434130000
C	20.963948000	-27.075063000	-0.189789000
N	15.183772000	-26.380461000	-2.176462000
C	19.143241000	-29.866293000	-0.466961000
C	18.579880000	-29.528508000	0.766443000
C	18.093863000	-30.521316000	1.621489000
C	18.164391000	-31.863711000	1.252320000
C	18.726708000	-32.210498000	0.020895000
C	19.213109000	-31.219012000	-0.828601000
C	22.064871000	-26.396014000	-1.018734000
O	21.842658000	-25.024988000	-1.244565000

H	16.049975000	-23.864483000	-1.773892000
H	18.362171000	-23.300440000	-1.070880000
H	17.189457000	-28.020283000	-1.578309000
H	19.071387000	-28.814194000	-2.347890000
H	20.684704000	-29.106701000	-1.752952000
H	20.688309000	-26.408142000	0.633209000
H	21.357051000	-27.996765000	0.252867000
H	14.913464000	-27.339341000	-2.013699000
H	14.440706000	-25.716433000	-2.017423000
H	18.523595000	-28.484037000	1.055882000
H	17.661194000	-30.241764000	2.577977000
H	17.788135000	-32.635308000	1.917532000
H	18.790019000	-33.253955000	-0.274597000
H	19.654862000	-31.497161000	-1.783371000
H	23.012327000	-26.495635000	-0.469684000
H	22.187312000	-26.956417000	-1.964625000
H	20.869905000	-24.896830000	-1.232710000

Rotamer Id

0 1

C	16.540644000	-24.789582000	-1.641047000
C	16.338799000	-26.141629000	-1.863378000
N	17.290282000	-27.080404000	-1.811584000
C	18.548008000	-26.681503000	-1.529898000
C	18.866424000	-25.326686000	-1.296666000
C	17.851265000	-24.365670000	-1.344280000
N	19.520476000	-27.671209000	-1.503340000
C	19.154463000	-29.086906000	-1.682301000
C	20.874209000	-27.403811000	-1.025851000
N	18.118157000	-23.036788000	-1.063569000
C	18.993942000	-29.860551000	-0.384489000
C	17.949836000	-29.562867000	0.504704000
C	17.809512000	-30.274709000	1.694396000
C	18.708113000	-31.296122000	2.016211000
C	19.746574000	-31.602481000	1.138245000
C	19.886388000	-30.886705000	-0.053621000
C	21.811914000	-26.917465000	-2.139533000
O	21.841765000	-27.814682000	-3.239920000
H	15.717209000	-24.084425000	-1.688640000
H	15.338481000	-26.503505000	-2.097736000
H	19.880913000	-25.011071000	-1.086552000
H	18.223646000	-29.103807000	-2.249527000
H	19.939776000	-29.554713000	-2.284553000
H	20.860499000	-26.683593000	-0.198077000
H	21.267910000	-28.338741000	-0.617698000
H	19.078603000	-22.739365000	-1.155818000
H	17.455420000	-22.363002000	-1.418179000
H	17.247073000	-28.776451000	0.247348000
H	16.994049000	-30.036525000	2.371788000
H	18.595705000	-31.849874000	2.943966000

H	20.448723000	-32.396055000	1.378090000
H	20.694697000	-31.132869000	-0.738646000
H	21.526744000	-25.906244000	-2.467170000
H	22.835472000	-26.861970000	-1.753427000
H	20.947391000	-27.818349000	-3.608719000

Rotamer Ie

0 1

C	16.903464407	-24.634291664	-1.132912928
C	16.630116260	-25.901136675	-1.643602498
N	17.526585257	-26.898800227	-1.701359804
C	18.799602607	-26.656956788	-1.248374973
C	19.173265959	-25.410764128	-0.709650716
C	18.210781210	-24.389848334	-0.658332368
N	19.725378537	-27.676361991	-1.442269173
C	19.259091075	-29.016891067	-1.837110405
C	20.964964307	-27.658989676	-0.649953781
N	18.555552580	-23.150039386	-0.165689997
C	18.943740136	-29.899260596	-0.656170808
C	17.797422907	-29.661027494	0.103283887
C	17.514334131	-30.461176713	1.206803400
C	18.370770647	-31.506942912	1.549222716
C	19.508777150	-31.753171463	0.783608775
C	19.796538131	-30.950825122	-0.319011850
C	22.050710305	-26.870833209	-1.412437302
O	22.444657911	-25.845346544	-0.510605297
H	16.143760718	-23.869412583	-1.106031351
H	15.633110764	-26.156876111	-2.027752841
H	20.183515696	-25.232091569	-0.358517165
H	18.352105000	-28.914179697	-2.491032512
H	20.048268680	-29.465226241	-2.484623197
H	20.810277157	-27.195602718	0.356465129
H	21.307296386	-28.696692128	-0.446295229
H	19.455253040	-23.001868895	0.234371306
H	17.869642116	-22.442411501	-0.037213801
H	17.125793891	-28.844169069	-0.173216726
H	16.622863965	-30.270118979	1.800918558
H	18.149661437	-32.131213239	2.411718270
H	20.173617890	-32.572523146	1.046676520
H	20.684010714	-31.150967014	-0.914485303
H	22.934450618	-27.491531077	-1.634028524
H	21.659653995	-26.422336717	-2.343124659
H	22.999608045	-25.184687806	-0.963595814

Rotamer If

0 1

C	16.706665932	-24.795993822	-1.275215178
C	17.978406876	-24.502054001	-0.793858155
N	18.986588486	-25.388652411	-0.743686787
C	18.757507887	-26.664979632	-1.197731591

C	17.497381048	-27.064947458	-1.687742362
C	16.465750809	-26.111803591	-1.732046840
N	19.861218387	-27.501382829	-1.204421922
C	19.711598997	-28.909005043	-1.578077624
C	21.023831533	-27.195842502	-0.351109195
N	15.224066989	-26.463199236	-2.205755940
C	19.141698426	-29.816874941	-0.515388682
C	18.533604681	-29.333376815	0.640917130
C	18.012753849	-30.221901340	1.580548088
C	18.097539844	-31.595505282	1.367800048
C	18.704171532	-32.081288287	0.209748826
C	19.224592879	-31.196160045	-0.729615727
C	22.072118200	-26.397011358	-1.156220198
O	21.963212651	-25.020056102	-0.885779312
H	15.932304527	-24.044797422	-1.294953101
H	18.229259577	-23.499677527	-0.418069711
H	17.314309195	-28.080809740	-2.003032665
H	19.079071104	-28.975142481	-2.500431367
H	20.721261445	-29.280441599	-1.891221501
H	20.716124262	-26.593264989	0.539654018
H	21.460982521	-28.134974602	0.047150792
H	15.041920067	-27.382064741	-2.540042835
H	14.486525384	-25.798460201	-2.255519245
H	18.453324207	-28.260762519	0.818528708
H	17.537283077	-29.837324692	2.481582011
H	17.691383373	-32.288143880	2.101255197
H	18.770645355	-33.154138810	0.040658298
H	19.695157425	-31.582278122	-1.630986561
H	23.102461167	-26.643089106	-0.844638843
H	21.960749228	-26.550162633	-2.244893329
H	21.006089316	-24.763287289	-0.876475704

Rotamer Ig

0 1

C	16.579553224	-24.834299287	-1.574332384
C	16.343413593	-26.169832829	-1.891240556
N	17.290209849	-27.120870938	-1.888970098
C	18.577159397	-26.760873036	-1.574898212
C	18.916988885	-25.437207302	-1.227423683
C	17.899443299	-24.466569942	-1.233454426
N	19.533633651	-27.757443718	-1.719988656
C	19.103216826	-29.165336637	-1.819710347
C	20.865449354	-27.562703298	-1.124276036
N	18.190928657	-23.161835793	-0.908305450
C	18.956246642	-29.820051431	-0.470095831
C	17.856793096	-29.516136736	0.333765251
C	17.731428550	-30.099807010	1.591530852
C	18.699368467	-30.995043647	2.045226180
C	19.790250064	-31.309256451	1.236985583
C	19.920000788	-30.723313038	-0.020731325

C	21.791108360	-26.911168032	-2.175223942
O	21.946991164	-27.768747604	-3.283875085
H	15.780195420	-24.110194730	-1.588812027
H	15.337968386	-26.522911862	-2.160500132
H	19.927066340	-25.163311138	-0.968527706
H	18.132777318	-29.216631742	-2.381482440
H	19.851103981	-29.699076405	-2.452833951
H	20.824557299	-26.950560730	-0.197893755
H	21.294201128	-28.546007905	-0.819575287
H	19.112333141	-22.886762376	-0.656436256
H	17.481739629	-22.465268624	-0.898855603
H	17.097666017	-28.818924406	-0.028756379
H	16.875860524	-29.857853740	2.218877857
H	18.601151304	-31.450426139	3.027950726
H	20.541414334	-32.013543489	1.587472966
H	20.771636607	-30.976113537	-0.649164116
H	21.400854346	-25.939694335	-2.526811341
H	22.819530639	-26.790577438	-1.790591893
H	21.060611262	-27.972132583	-3.657653456

Rotamer II

0 1

C	3.384922000	-2.002774000	0.205755000
C	2.218979000	-2.266164000	0.905308000
N	1.164348000	-1.446934000	0.979943000
C	1.243545000	-0.265013000	0.333073000
C	2.392654000	0.111907000	-0.395695000
C	3.474670000	-0.770011000	-0.471568000
N	0.138530000	0.563660000	0.424659000
C	-1.008983000	0.203090000	1.263437000
C	0.034414000	1.798514000	-0.333575000
N	4.592666000	-0.459151000	-1.227550000
C	-2.210614000	-0.297673000	0.480274000
C	-2.132815000	-1.492927000	-0.250654000
C	-3.234214000	-1.956342000	-0.967002000
C	-4.431477000	-1.234153000	-0.965842000
C	-4.519621000	-0.046608000	-0.241510000
C	-3.413307000	0.416635000	0.475761000
C	0.633625000	3.004470000	0.396120000
O	0.454994000	4.132842000	-0.450795000
H	4.196650000	-2.722405000	0.176861000
H	2.117690000	-3.207391000	1.444364000
H	2.457889000	1.071845000	-0.893662000
H	-0.666309000	-0.564997000	1.957287000
H	-1.301233000	1.084055000	1.848857000
H	0.512843000	1.693135000	-1.312115000
H	-1.023356000	1.997249000	-0.531550000
H	4.736123000	0.517771000	-1.438744000
H	5.444086000	-0.944359000	-0.985295000
H	-1.203886000	-2.055245000	-0.241748000

H	-3.161902000	-2.885410000	-1.525715000
H	-5.288633000	-1.597491000	-1.525558000
H	-5.445465000	0.521798000	-0.234650000
H	-3.487548000	1.342115000	1.042720000
H	0.123630000	3.135543000	1.363493000
H	1.697042000	2.818831000	0.610959000
H	0.792960000	4.911479000	0.008221000

Rotamer III

0 1

C	3.385810000	-2.001804000	0.206009000
C	2.220025000	-2.265599000	0.905665000
N	1.165191000	-1.446667000	0.980543000
C	1.243969000	-0.264615000	0.333863000
C	2.392971000	0.112798000	-0.394841000
C	3.475175000	-0.768879000	-0.471063000
N	0.138670000	0.563662000	0.425607000
C	-1.008882000	0.202220000	1.263930000
C	0.033444000	1.797847000	-0.333539000
N	4.592980000	-0.457615000	-1.227172000
C	-2.210217000	-0.298458000	0.480275000
C	-2.130796000	-1.491257000	-0.254488000
C	-3.232011000	-1.954584000	-0.971176000
C	-4.430722000	-1.234828000	-0.966488000
C	-4.520501000	-0.049777000	-0.238284000
C	-3.414353000	0.413428000	0.479260000
C	0.631199000	3.004969000	0.395411000
O	0.451951000	4.132421000	-0.452593000
H	4.197669000	-2.721271000	0.176843000
H	2.119036000	-3.206945000	1.444572000
H	2.458052000	1.072835000	-0.892647000
H	-0.666035000	-0.566214000	1.957327000
H	-1.301531000	1.082684000	1.849915000
H	0.512364000	1.692348000	-1.311819000
H	-1.024491000	1.995217000	-0.532016000
H	4.736188000	0.519373000	-1.438237000
H	5.444558000	-0.942678000	-0.985183000
H	-1.200786000	-2.051826000	-0.248310000
H	-3.158409000	-2.881723000	-1.532920000
H	-5.287713000	-1.598108000	-1.526489000
H	-5.447455000	0.516779000	-0.228689000
H	-3.489823000	1.337034000	1.049107000
H	0.120697000	3.136479000	1.362461000
H	1.694687000	2.820409000	0.610852000
H	0.788830000	4.911813000	0.005938000

Rotamer IV

0 1

C	3.385459000	-2.002689000	0.206087000
C	2.219143000	-2.266335000	0.904912000

N	1.164324000	-1.447289000	0.978992000
C	1.243728000	-0.265274000	0.332328000
C	2.393256000	0.111944000	-0.395659000
C	3.475404000	-0.769844000	-0.471070000
N	0.138483000	0.563142000	0.423243000
C	-1.008794000	0.203071000	1.262528000
C	0.034858000	1.798401000	-0.334393000
N	4.593771000	-0.458750000	-1.226420000
C	-2.210818000	-0.297527000	0.479921000
C	-2.133461000	-1.492833000	-0.251009000
C	-3.235275000	-1.956191000	-0.966751000
C	-4.432504000	-1.233942000	-0.964954000
C	-4.520226000	-0.046395000	-0.240584000
C	-3.413489000	0.416815000	0.476066000
C	0.633393000	3.004090000	0.396301000
O	0.454847000	4.132873000	-0.450085000
H	4.197302000	-2.722201000	0.177576000
H	2.117693000	-3.207626000	1.443825000
H	2.458682000	1.071985000	-0.893405000
H	-0.666082000	-0.565009000	1.956369000
H	-1.300567000	1.084191000	1.847971000
H	0.514126000	1.693530000	-1.312560000
H	-1.022775000	1.997147000	-0.533121000
H	4.737115000	0.518194000	-1.437590000
H	5.445174000	-0.943747000	-0.983685000
H	-1.204552000	-2.055209000	-0.242581000
H	-3.163334000	-2.885279000	-1.525481000
H	-5.289973000	-1.597271000	-1.524197000
H	-5.446050000	0.522037000	-0.233218000
H	-3.487405000	1.342283000	1.043092000
H	0.122917000	3.134495000	1.363506000
H	1.696761000	2.818633000	0.611518000
H	0.792106000	4.911411000	0.009618000

Rotamer V

1 1

C	-1.845934000	-2.967832000	-0.234768000
C	-0.597039000	-2.634348000	-0.648891000
N	-0.152052000	-1.343738000	-0.606585000
C	-0.924810000	-0.308595000	-0.133477000
C	-2.220998000	-0.616811000	0.303975000
C	-2.700084000	-1.934854000	0.269094000
N	-0.378656000	0.934156000	-0.123612000
C	0.874392000	1.222339000	-0.851660000
C	-1.116048000	2.069670000	0.444268000
N	-3.944687000	-2.240110000	0.684866000
C	2.124202000	0.569058000	-0.277236000
C	2.374445000	0.562036000	1.102519000
C	3.547367000	-0.001610000	1.600692000
C	4.483646000	-0.565214000	0.728830000

C	4.242191000	-0.564913000	-0.644229000
C	3.065302000	-0.001964000	-1.145785000
C	-2.052184000	2.763126000	-0.552546000
O	-2.764170000	3.716913000	0.207831000
H	-2.180028000	-3.996786000	-0.284567000
H	0.111515000	-3.359762000	-1.028955000
H	-2.870855000	0.174332000	0.649725000
H	0.759391000	0.938441000	-1.905840000
H	0.984335000	2.309639000	-0.839273000
H	-1.692617000	1.739231000	1.311251000
H	-0.387518000	2.792418000	0.818324000
H	-4.563441000	-1.536184000	1.055781000
H	-4.289145000	-3.186426000	0.651807000
H	1.652122000	0.997742000	1.787085000
H	3.734383000	0.001681000	2.669904000
H	5.397031000	-1.001082000	1.120666000
H	4.966366000	-0.997662000	-1.327085000
H	2.889282000	0.010973000	-2.219382000
H	-1.460560000	3.224617000	-1.357797000
H	-2.717993000	2.020913000	-1.021819000
H	-3.246704000	4.304196000	-0.387614000
H	0.828874000	-1.164276000	-0.802266000

Rotamer VI

1 1

C	-1.841000000	-2.968823000	-0.235845000
C	-0.592095000	-2.633271000	-0.648280000
N	-0.148972000	-1.342084000	-0.604498000
C	-0.923699000	-0.308369000	-0.131496000
C	-2.219983000	-0.618734000	0.304169000
C	-2.697183000	-1.937423000	0.267728000
N	-0.379214000	0.935184000	-0.119881000
C	0.873379000	1.226189000	-0.847707000
C	-1.120279000	2.069391000	0.445928000
N	-3.941886000	-2.244665000	0.681743000
C	2.123954000	0.572230000	-0.275807000
C	2.375720000	0.562569000	1.103668000
C	3.549372000	-0.001710000	1.599408000
C	4.484867000	-0.563321000	0.725411000
C	4.241870000	-0.560448000	-0.647374000
C	3.064269000	0.003165000	-1.146506000
C	-2.057029000	2.759397000	-0.552700000
O	-2.773846000	3.710745000	0.206213000
H	-2.173589000	-3.998205000	-0.286793000
H	0.117923000	-3.357403000	-1.028057000
H	-2.871394000	0.171191000	0.649738000
H	0.758184000	0.945222000	-1.902669000
H	0.982708000	2.313504000	-0.832148000
H	-1.697202000	1.738270000	1.312426000
H	-0.394104000	2.794286000	0.820358000

H	-4.562147000	-1.541797000	1.052151000
H	-4.285084000	-3.191397000	0.647480000
H	1.653995000	0.996686000	1.789873000
H	3.737570000	-0.000471000	2.668417000
H	5.398810000	-0.999677000	1.115400000
H	4.965403000	-0.991649000	-1.331888000
H	2.887012000	0.018136000	-2.219868000
H	-1.465697000	3.222915000	-1.356989000
H	-2.719301000	2.014634000	-1.022926000
H	-3.256713000	4.296730000	-0.390238000
H	0.831921000	-1.161038000	-0.799104000

Rotamer VII

1 1

C	-1.846126000	-2.967533000	-0.235205000
C	-0.596868000	-2.634074000	-0.648270000
N	-0.151766000	-1.343550000	-0.605207000
C	-0.924771000	-0.308438000	-0.132420000
C	-2.221311000	-0.616616000	0.303994000
C	-2.700539000	-1.934607000	0.268319000
N	-0.378416000	0.934251000	-0.121929000
C	0.874412000	1.222614000	-0.850248000
C	-1.116433000	2.069834000	0.445017000
N	-3.945525000	-2.239830000	0.682953000
C	2.124439000	0.568967000	-0.276718000
C	2.374906000	0.560189000	1.103000000
C	3.548294000	-0.003355000	1.600193000
C	4.484869000	-0.565016000	0.727409000
C	4.243189000	-0.562981000	-0.645614000
C	3.065780000	-0.000240000	-1.146186000
C	-2.052512000	2.762379000	-0.552506000
O	-2.765976000	3.715533000	0.207292000
H	-2.180317000	-3.996425000	-0.285613000
H	0.111884000	-3.359461000	-1.028017000
H	-2.871392000	0.174496000	0.649402000
H	0.759009000	0.939334000	-1.904563000
H	0.984554000	2.309889000	-0.837299000
H	-1.693324000	1.739691000	1.311904000
H	-0.388300000	2.793004000	0.819027000
H	-4.564576000	-1.535914000	1.053390000
H	-4.290142000	-3.186062000	0.649124000
H	1.652431000	0.994496000	1.788290000
H	3.735452000	-0.001438000	2.669385000
H	5.398633000	-1.000752000	1.118504000
H	4.967562000	-0.994250000	-1.329193000
H	2.889533000	0.014010000	-2.219724000
H	-1.460871000	3.224402000	-1.357435000
H	-2.717271000	2.019432000	-1.022069000
H	-3.248379000	4.302503000	-0.388567000
H	0.829308000	-1.164050000	-0.800185000

Rotamer VIII

1 1

C	-1.842199000	-2.968364000	-0.237017000
C	-0.593526000	-2.633002000	-0.650329000
N	-0.149971000	-1.341973000	-0.606355000
C	-0.923946000	-0.308230000	-0.132192000
C	-2.219954000	-0.618401000	0.304418000
C	-2.697648000	-1.936910000	0.267728000
N	-0.379019000	0.935098000	-0.120331000
C	0.873781000	1.225676000	-0.847873000
C	-1.119458000	2.069475000	0.445961000
N	-3.942076000	-2.243943000	0.682702000
C	2.124026000	0.571390000	-0.275565000
C	2.374223000	0.559552000	1.104174000
C	3.547776000	-0.004614000	1.600284000
C	4.484751000	-0.563892000	0.726396000
C	4.243302000	-0.558862000	-0.646661000
C	3.065760000	0.004539000	-1.146159000
C	-2.056290000	2.759959000	-0.552264000
O	-2.772012000	3.711961000	0.206848000
H	-2.175141000	-3.997626000	-0.288132000
H	0.115952000	-3.357189000	-1.031006000
H	-2.870685000	0.171534000	0.651247000
H	0.758832000	0.944683000	-1.902858000
H	0.983427000	2.312973000	-0.832458000
H	-1.696079000	1.738543000	1.312733000
H	-0.392845000	2.794082000	0.820100000
H	-4.561740000	-1.541054000	1.054066000
H	-4.285502000	-3.190595000	0.648551000
H	1.651328000	0.991835000	1.790297000
H	3.734735000	-0.005072000	2.669512000
H	5.398630000	-1.000127000	1.116668000
H	4.967957000	-0.988302000	-1.331094000
H	2.889686000	0.021109000	-2.219690000
H	-1.464983000	3.222856000	-1.356931000
H	-2.719364000	2.015643000	-1.022050000
H	-3.255006000	4.297996000	-0.389452000
H	0.830772000	-1.161161000	-0.801809000

Rotamer IX

1 1

C	4.151678000	-0.322601000	0.788067000
C	3.229255000	0.390802000	1.522591000
N	1.928792000	0.492446000	1.194622000
C	1.570164000	-0.144657000	0.103316000
C	2.377815000	-0.894711000	-0.725308000
C	3.741733000	-0.996812000	-0.379401000
N	0.123137000	-0.020376000	-0.240486000
C	-0.789709000	-0.319010000	0.970679000

C	-0.210862000	1.266346000	-0.977744000
N	4.610845000	-1.710565000	-1.130731000
C	-2.202851000	-0.580466000	0.540442000
C	-3.185403000	0.403812000	0.665928000
C	-4.490743000	0.148309000	0.260619000
C	-4.823808000	-1.091187000	-0.275771000
C	-3.853280000	-2.081731000	-0.397155000
C	-2.550522000	-1.829153000	0.014567000
C	0.093577000	2.556323000	-0.222982000
O	-0.524086000	3.551922000	-1.023923000
H	5.182479000	-0.364532000	1.110937000
H	3.533319000	0.910182000	2.421651000
H	1.992989000	-1.387953000	-1.608497000
H	-0.340981000	-1.189213000	1.443077000
H	-0.690151000	0.521482000	1.645639000
H	0.346138000	1.239837000	-1.911508000
H	-1.273364000	1.222890000	-1.204344000
H	4.319433000	-2.186910000	-1.964317000
H	5.579157000	-1.778123000	-0.875277000
H	-2.936396000	1.367205000	1.092023000
H	-5.246200000	0.913915000	0.367948000
H	-5.839656000	-1.289707000	-0.587758000
H	-4.114638000	-3.052110000	-0.795359000
H	-1.810129000	-2.618946000	-0.048076000
H	-0.327091000	2.538025000	0.785179000
H	1.168173000	2.715058000	-0.135716000
H	-0.249051000	4.424078000	-0.727257000
H	-0.083025000	-0.762688000	-0.907899000

Rotamer X

1 1

C	17.191741000	-25.638229000	-3.339708000
C	17.666123000	-26.924229000	-3.156082000
N	18.400295000	-27.310990000	-2.093047000
C	18.649179000	-26.365028000	-1.207220000
C	18.243509000	-25.043165000	-1.253498000
C	17.475318000	-24.643481000	-2.374300000
N	19.459528000	-26.821820000	-0.038948000
C	18.883401000	-28.112917000	0.595854000
C	20.952812000	-26.874231000	-0.329089000
N	17.031560000	-23.372777000	-2.511330000
C	19.467862000	-28.359030000	1.957167000
C	20.510431000	-29.278570000	2.139604000
C	21.047585000	-29.489303000	3.409505000
C	20.548901000	-28.782909000	4.505360000
C	19.505504000	-27.870059000	4.334627000
C	18.964365000	-27.662513000	3.067147000
C	21.379200000	-27.824811000	-1.448710000
O	22.790604000	-27.810475000	-1.349186000
H	16.603936000	-25.396521000	-4.218942000

H	17.452673000	-27.694747000	-3.891090000
H	18.497895000	-24.331546000	-0.473200000
H	17.806783000	-27.937315000	0.636692000
H	19.070440000	-28.911686000	-0.117711000
H	21.257157000	-25.854121000	-0.571240000
H	21.437762000	-27.175935000	0.600806000
H	17.237814000	-22.663078000	-1.827551000
H	16.485691000	-23.098279000	-3.312084000
H	20.894506000	-29.839492000	1.291756000
H	21.850010000	-30.207392000	3.543080000
H	20.965295000	-28.950689000	5.493412000
H	19.105301000	-27.333408000	5.188680000
H	18.129047000	-26.975101000	2.946218000
H	20.963952000	-28.830814000	-1.295072000
H	21.020628000	-27.466979000	-2.420085000
H	23.164432000	-28.250122000	-2.123465000
H	19.359631000	-26.110610000	0.690885000

Rotamer XI

1 1

C	18.655013000	-23.633293000	-2.215946000
C	19.695381000	-24.462747000	-2.592995000
N	19.858246000	-25.721036000	-2.136024000
C	18.940228000	-26.132568000	-1.282003000
C	17.848679000	-25.422211000	-0.815152000
C	17.685731000	-24.100380000	-1.297098000
N	19.142655000	-27.530068000	-0.795900000
C	19.372884000	-28.521527000	-1.964549000
C	20.164109000	-27.631649000	0.328016000
N	16.650560000	-23.324957000	-0.900211000
C	19.218000000	-29.944817000	-1.511149000
C	20.338775000	-30.736514000	-1.223410000
C	20.177361000	-32.054245000	-0.795496000
C	18.896582000	-32.590130000	-0.650594000
C	17.774155000	-31.811393000	-0.941907000
C	17.934271000	-30.496306000	-1.374696000
C	21.580803000	-27.170872000	-0.017736000
O	22.322962000	-27.563470000	1.121234000
H	18.583622000	-22.631164000	-2.625374000
H	20.443562000	-24.113462000	-3.298487000
H	17.141280000	-25.845827000	-0.107960000
H	18.622923000	-28.243011000	-2.707282000
H	20.354809000	-28.290054000	-2.370028000
H	19.772147000	-27.039052000	1.156924000
H	20.191813000	-28.680994000	0.626358000
H	15.963629000	-23.654709000	-0.242064000
H	16.543233000	-22.386098000	-1.248857000
H	21.338284000	-30.327849000	-1.345673000
H	21.050171000	-32.662164000	-0.580988000
H	18.772648000	-33.616723000	-0.321160000

H	16.778626000	-32.232571000	-0.846564000
H	17.057034000	-29.905632000	-1.632560000
H	21.943263000	-27.656097000	-0.934939000
H	21.607350000	-26.088795000	-0.186763000
H	23.201983000	-27.166273000	1.075424000
H	18.259406000	-27.831103000	-0.374368000

Rotamer XII

1 1

C	18.689601000	-23.411976000	-1.644335000
C	19.847847000	-24.092446000	-1.972945000
N	20.027958000	-25.413447000	-1.770062000
C	19.005061000	-26.043197000	-1.223690000
C	17.790047000	-25.500954000	-0.844570000
C	17.609084000	-24.112532000	-1.058092000
N	19.230107000	-27.504659000	-1.014528000
C	19.758019000	-28.198518000	-2.295832000
C	20.028232000	-27.813937000	0.244142000
N	16.456745000	-23.488951000	-0.720976000
C	19.634581000	-29.691975000	-2.197342000
C	20.738728000	-30.486796000	-1.858370000
C	20.604860000	-31.871923000	-1.762993000
C	19.368167000	-32.472830000	-2.004004000
C	18.264197000	-31.689897000	-2.349527000
C	18.397903000	-30.306283000	-2.450231000
C	21.439710000	-27.227498000	0.296679000
O	21.987630000	-27.836659000	1.450326000
H	18.611670000	-22.347330000	-1.837842000
H	20.682129000	-23.563504000	-2.424266000
H	17.001657000	-26.101017000	-0.399212000
H	19.143474000	-27.787660000	-3.099016000
H	20.777338000	-27.846297000	-2.434021000
H	19.438855000	-27.440787000	1.083955000
H	20.089588000	-28.901418000	0.311646000
H	15.689421000	-23.985891000	-0.299039000
H	16.337445000	-22.501097000	-0.877316000
H	21.706381000	-30.025029000	-1.681126000
H	21.465840000	-32.480355000	-1.506280000
H	19.266454000	-33.550985000	-1.933169000
H	17.306677000	-32.157622000	-2.554347000
H	17.542414000	-29.705005000	-2.752727000
H	22.007066000	-27.476062000	-0.611277000
H	21.404253000	-26.135162000	0.372319000
H	22.822250000	-27.402550000	1.668277000
H	18.312173000	-27.928697000	-0.853099000

Rotamer XIII

1 1

C	1.918214000	3.083645000	0.447724000
C	2.647133000	1.958686000	0.854262000

N	2.270134000	0.703346000	0.649471000
C	1.106308000	0.444601000	0.009427000
C	0.270786000	1.512976000	-0.442731000
C	0.723524000	2.788968000	-0.195912000
N	0.762533000	-0.855907000	-0.173091000
C	-0.403712000	-1.219911000	-0.982661000
C	1.604899000	-1.933957000	0.366787000
N	-0.140273000	3.928893000	-0.644270000
C	-1.758429000	-0.932843000	-0.346874000
C	-1.950193000	-0.999980000	1.038126000
C	-3.215185000	-0.784376000	1.586564000
C	-4.302703000	-0.500207000	0.758246000
C	-4.119416000	-0.429992000	-0.623614000
C	-2.852964000	-0.641407000	-1.171660000
C	2.704808000	-2.379595000	-0.599475000
O	3.341776000	-3.474088000	0.030685000
H	2.271875000	4.091189000	0.634103000
H	3.592048000	2.093070000	1.375680000
H	-0.691229000	1.322299000	-0.903029000
H	-0.343797000	-0.723338000	-1.960373000
H	-0.314677000	-2.290639000	-1.188001000
H	2.063026000	-1.598945000	1.296976000
H	0.958705000	-2.786734000	0.590317000
H	-1.054702000	3.592128000	-0.965626000
H	-0.302827000	4.591354000	0.123351000
H	-1.110519000	-1.222126000	1.690060000
H	-3.352232000	-0.845308000	2.661906000
H	-5.286978000	-0.339268000	1.186713000
H	-4.960855000	-0.215304000	-1.275478000
H	-2.718950000	-0.597311000	-2.250646000
H	2.260563000	-2.662266000	-1.568419000
H	3.392091000	-1.539535000	-0.776726000
H	4.085340000	-3.760233000	-0.514237000
H	0.293729000	4.449712000	-1.416067000

Rotamer XIV

1 1

C	1.914319000	3.085300000	0.448034000
C	2.644282000	1.961203000	0.855031000
N	2.268786000	0.705420000	0.650162000
C	1.105539000	0.445289000	0.009650000
C	0.269080000	1.512706000	-0.443097000
C	0.720310000	2.789210000	-0.196232000
N	0.763289000	-0.855618000	-0.172906000
C	-0.402440000	-1.220936000	-0.982615000
C	1.607080000	-1.932720000	0.366675000
N	-0.144548000	3.928184000	-0.644949000
C	-1.757412000	-0.934879000	-0.346943000
C	-1.949092000	-1.002354000	1.038056000
C	-3.214117000	-0.787245000	1.586599000

C	-4.301767000	-0.503223000	0.758397000
C	-4.118574000	-0.432691000	-0.623458000
C	-2.852081000	-0.643629000	-1.171616000
C	2.707476000	-2.376715000	-0.599783000
O	3.346521000	-3.469924000	0.030488000
H	2.266723000	4.093261000	0.634533000
H	3.588815000	2.096679000	1.376858000
H	-0.692458000	1.320977000	-0.903964000
H	-0.342871000	-0.724563000	-1.960456000
H	-0.312380000	-2.291625000	-1.187668000
H	2.064934000	-1.597271000	1.296837000
H	0.962023000	-2.786373000	0.590163000
H	-1.056404000	3.589974000	-0.972036000
H	-0.312795000	4.587608000	0.124074000
H	-1.109301000	-1.224303000	1.689915000
H	-3.351091000	-0.848420000	2.661936000
H	-5.286059000	-0.342621000	1.186951000
H	-4.960110000	-0.218119000	-1.275239000
H	-2.718134000	-0.599219000	-2.250599000
H	2.263470000	-2.660419000	-1.568522000
H	3.393208000	-1.535468000	-0.777420000
H	4.090064000	-3.755328000	-0.514851000
H	0.291887000	4.452817000	-1.412773000

Rotamer XV

1 1

C	1.907973000	3.088339000	0.447630000
C	2.640170000	1.965797000	0.854903000
N	2.267330000	0.709200000	0.650116000
C	1.104765000	0.446526000	0.009403000
C	0.266428000	1.512192000	-0.444029000
C	0.714891000	2.789661000	-0.197138000
N	0.765018000	-0.855131000	-0.172560000
C	-0.400327000	-1.223030000	-0.981681000
C	1.611113000	-1.930302000	0.367211000
N	-0.152278000	3.926744000	-0.646193000
C	-1.755613000	-0.937821000	-0.346317000
C	-1.947151000	-1.002994000	1.038806000
C	-3.212516000	-0.788864000	1.586962000
C	-4.300628000	-0.508083000	0.758274000
C	-4.117565000	-0.439814000	-0.623717000
C	-2.850772000	-0.649810000	-1.171503000
C	2.712194000	-2.372377000	-0.599376000
O	3.353199000	-3.464497000	0.030783000
H	2.258180000	4.097050000	0.634196000
H	3.584305000	2.103295000	1.376924000
H	-0.694466000	1.318585000	-0.905491000
H	-0.341511000	-0.727929000	-1.960221000
H	-0.308944000	-2.293884000	-1.185278000
H	2.068438000	-1.593673000	1.297201000

H	0.967845000	-2.785212000	0.591014000
H	-1.061986000	3.586232000	-0.976843000
H	-0.325099000	4.584054000	0.123635000
H	-1.107037000	-1.222461000	1.691079000
H	-3.349374000	-0.848294000	2.662413000
H	-5.285172000	-0.348289000	1.186550000
H	-4.959451000	-0.227806000	-1.275884000
H	-2.716935000	-0.607229000	-2.250573000
H	2.268513000	-2.656799000	-1.568050000
H	3.396481000	-1.529971000	-0.777100000
H	4.097487000	-3.748264000	-0.514393000
H	0.284863000	4.454361000	-1.411559000

Rotamer XVI

1 1

C	1.909579000	3.087432000	0.448082000
C	2.641072000	1.964547000	0.855670000
N	2.267477000	0.708144000	0.651141000
C	1.104780000	0.446029000	0.010442000
C	0.267077000	1.512082000	-0.443241000
C	0.716338000	2.789336000	-0.196671000
N	0.764345000	-0.855475000	-0.171421000
C	-0.400795000	-1.222845000	-0.981090000
C	1.610635000	-1.931044000	0.367329000
N	-0.149875000	3.926866000	-0.646435000
C	-1.756233000	-0.937240000	-0.346244000
C	-1.948711000	-1.004532000	1.038653000
C	-3.214202000	-0.790027000	1.586356000
C	-4.301480000	-0.506746000	0.757403000
C	-4.117465000	-0.436339000	-0.624345000
C	-2.850517000	-0.646694000	-1.171668000
C	2.711137000	-2.372580000	-0.600147000
O	3.352478000	-3.465127000	0.028922000
H	2.260420000	4.095971000	0.634389000
H	3.585275000	2.101587000	1.377686000
H	-0.693799000	1.318842000	-0.904855000
H	-0.341440000	-0.727672000	-1.959558000
H	-0.309729000	-2.293719000	-1.184704000
H	2.068468000	-1.594944000	1.297260000
H	0.967426000	-2.786026000	0.591030000
H	-1.059889000	3.586936000	-0.976848000
H	-0.322094000	4.584871000	0.122934000
H	-1.109142000	-1.225851000	1.691021000
H	-3.351828000	-0.851091000	2.661615000
H	-5.286136000	-0.346642000	1.185309000
H	-4.958719000	-0.222347000	-1.276679000
H	-2.715908000	-0.602462000	-2.250576000
H	2.266899000	-2.656369000	-1.568761000
H	3.395345000	-1.530074000	-0.777727000
H	4.096408000	-3.748587000	-0.516903000

H	0.287713000	4.453562000	-1.412179000

Adduct XVIIa

1 1

C	3.62119	-1.04846	-0.25570
C	2.59388	-1.04100	-1.15785
N	1.52358	-0.14214	-1.05997
C	1.46753	0.70186	0.07051
C	2.46648	0.67633	1.03063
C	3.57565	-0.17916	0.87427
N	0.40299	1.63704	0.21826
C	-0.72918	1.20611	1.04729
C	0.03716	2.32748	-1.03963
N	4.56830	-0.25016	1.86156
C	-2.15049	1.40436	0.57869
C	-2.50998	1.80827	-0.70511
C	-3.85404	1.96536	-1.04537
C	-4.85008	1.71235	-0.10700
C	-4.49738	1.30344	1.17921
C	-3.15778	1.15132	1.51980
C	1.07995	3.42504	-1.33392
O	2.07110	2.94421	-2.21552
H	4.46323	-1.72174	-0.37862
H	2.55809	-1.70089	-2.02537
H	2.38343	1.31501	1.90759
H	-0.57885	0.12245	1.28961
H	-0.60002	1.74995	2.02249
H	-0.01094	1.63910	-1.91355
H	-0.96995	2.78141	-0.92844
H	4.65005	0.54556	2.46419
H	5.45637	-0.60773	1.56817
H	-1.75117	2.00033	-1.46606
H	-4.12023	2.28869	-2.05158
H	-5.89734	1.83998	-0.37132
H	-5.27214	1.11146	1.91966
H	-2.89183	0.84083	2.52893
H	0.63615	4.26750	-1.89420
H	1.56119	3.79870	-0.41251
H	2.47437	2.12268	-1.85951
O	-0.70708	-1.67477	-2.35714
H	0.65615	-0.41249	-1.52023
H	-1.45831	-1.18656	-2.70559
H	-1.06691	-2.45895	-1.94947

Adduct XVIIb

1 1

C	3.64432	-0.79763	-0.46307
C	2.55138	-0.92460	-1.30824
N	1.31291	-0.28020	-1.03591
C	1.27770	0.60235	0.07489

C	2.39667	0.77025	0.87917
C	3.62232	0.11003	0.61715
N	0.13646	1.43901	0.28309
C	-1.08310	0.67679	0.71728
C	0.16366	2.62745	-0.60072
N	4.70005	0.24616	1.46577
C	-2.43048	1.28043	0.40109
C	-3.45279	0.58534	-0.29755
C	-4.68414	1.18268	-0.52118
C	-5.04736	2.37657	0.15738
C	-4.06347	3.00718	0.93804
C	-2.71845	2.51676	0.98111
C	1.53930	3.36070	-0.38241
O	2.44785	2.88854	-1.33148
H	4.53348	-1.40324	-0.70462
H	2.59598	-1.52882	-2.27155
H	2.27006	1.50940	1.66653
H	-1.03523	-0.40325	0.37327
H	-1.04517	0.68274	1.85458
H	0.18816	2.39493	-1.62162
H	-0.63352	3.29863	-0.29261
H	4.76017	1.08487	2.00944
H	5.58617	0.01839	1.03225
H	-3.19508	-0.37312	-0.75994
H	-5.32745	0.70382	-1.26870
H	-6.05808	2.73622	-0.04856
H	-4.26898	3.97116	1.46890
H	-1.90771	3.18363	1.24902
H	1.38249	4.42576	-0.58440
H	1.85641	3.19725	0.65278
H	2.77319	1.98519	-1.09619
O	0.02107	-3.26274	-3.36529
H	0.60463	-0.22241	-1.72889
H	0.24015	-2.49315	-2.83686
H	0.57734	-3.94747	-3.03831

Adduct XVIIIa

1 1

C	0.99277	-3.24308	-1.94808
C	0.71184	-3.37751	-0.53601
N	0.03027	-2.53718	0.17135
C	-0.56848	-1.37310	-0.47315
C	0.19619	-0.89285	-1.75421
C	0.71982	-2.05016	-2.55001
N	-0.56446	-0.18638	0.42797
C	-1.32181	-0.30287	1.68762
C	-0.86519	1.05343	-0.31942
N	0.93783	-1.82720	-3.88628
C	-2.41117	0.71822	1.90787
C	-3.70663	0.47762	1.44817

C	-4.70783	1.42575	1.64406
C	-4.42059	2.61805	2.30686
C	-3.13202	2.85429	2.78260
C	-2.13073	1.90508	2.58855
C	-1.13344	2.17534	0.70601
O	-1.12106	3.42661	0.05218
H	1.40872	-4.08877	-2.47098
H	1.10150	-4.26662	-0.01659
H	-0.46529	-0.23150	-2.34259
H	-1.74850	-1.33656	1.74388
H	-0.56975	-0.25073	2.51163
H	0.00481	1.35218	-0.94628
H	-1.72212	0.95529	-1.02123
H	0.78193	-0.93536	-4.29929
H	1.33261	-2.53565	-4.46529
H	-3.93147	-0.46213	0.93297
H	-5.71702	1.22994	1.28564
H	-5.20273	3.35786	2.46252
H	-2.90863	3.77891	3.31120
H	-1.13003	2.08940	2.97446
H	-2.09376	2.04735	1.23073
H	-0.31504	2.26311	1.44440
H	-1.76688	3.44408	-0.68022
H	1.05046	-0.24990	-1.43264
O	-3.88127	-1.68371	0.53428
H	-1.61923	-1.67086	-0.77029
H	-2.92062	-1.70852	0.55293
H	-4.14553	-1.90057	1.43101

Adduct XVIIIb

1 1

C	-1.60477	-2.33444	-0.55359
C	-2.57454	-1.25009	-0.73339
N	-2.32931	-0.00951	-0.99614
C	-0.94756	0.43944	-1.18363
C	0.01426	-0.72227	-1.43468
C	-0.30009	-1.97050	-0.65754
N	-0.35742	1.32047	-0.11181
C	-0.47424	0.93846	1.33523
C	-0.95063	2.60947	-0.27515
N	0.69836	-2.74446	-0.04253
C	-1.54729	1.49880	2.17835
C	-2.03869	2.81488	2.43332
C	-3.30936	3.02613	3.00593
C	-4.08837	1.90971	3.38859
C	-3.37508	0.69350	3.46606
C	-2.17281	0.44031	2.80533
C	-0.91203	3.40132	-1.62222
O	-0.27816	2.70189	-2.59939
H	-1.90660	-3.32638	-0.21548

H	-3.62163	-1.46221	-0.45489
H	0.12095	-0.96516	-2.49315
H	-0.59995	-0.18041	1.51217
H	0.57921	1.22362	1.63349
H	-2.05146	2.61881	0.02032
H	-0.25569	3.28306	0.09531
H	1.65073	-2.51934	-0.22408
H	0.52471	-3.54187	0.52323
H	-1.38510	3.62746	2.01927
H	-3.77358	3.95171	3.05655
H	-5.09450	2.05365	3.62993
H	-3.85536	-0.17777	3.80395
H	-1.82744	-0.62801	2.67934
H	-2.02728	3.45557	-1.80250
H	-0.35341	4.32123	-1.55189
H	0.64146	2.60270	-2.35329
H	1.05480	-0.36124	-1.18470
O	-1.04365	-10.36667	-2.18387
H	-1.02948	1.04718	-2.15179
H	-0.99773	-10.35549	-3.10468
H	-1.66544	-11.05041	-2.01732

Adduct XIXa

1 1

C	3.16094	-1.66078	-0.38964
C	2.75238	-1.67435	0.94082
N	1.76443	-0.90913	1.43319
C	1.12647	-0.04388	0.57997
C	1.46286	0.04145	-0.78362
C	2.49194	-0.78172	-1.27213
N	0.19923	0.81793	1.15946
C	-0.94628	0.25934	1.89845
C	-0.05688	2.11123	0.50629
N	2.82855	-0.74255	-2.60386
C	-2.25523	0.91237	1.53356
C	-2.84480	0.64527	0.29721
C	-4.04279	1.26283	-0.05129
C	-4.65995	2.14173	0.83809
C	-4.07802	2.39937	2.07780
C	-2.87625	1.78589	2.42689
C	-0.09099	3.22492	1.57416
O	0.88775	4.16060	1.14190
H	3.22734	-2.33841	1.67559
H	0.95189	0.73076	-1.44611
H	-1.00391	-0.84576	1.70788
H	-0.72383	0.36659	2.98560
H	0.72333	2.35554	-0.25747
H	-1.01631	2.07854	-0.05359
H	2.39710	-0.08399	-3.21246
H	3.60318	-1.27105	-2.95445

H	-2.36021	-0.04936	-0.39394
H	-4.49747	1.05783	-1.01839
H	-5.59524	2.62447	0.56522
H	-4.56174	3.08039	2.77393
H	-2.42928	1.98890	3.39707
H	-1.06693	3.73601	1.60913
H	0.17381	2.84445	2.57699
H	1.04395	4.83478	1.82777
O	5.18791	-2.38801	-3.48890
H	4.98992	-3.05154	-4.27439
H	6.05255	-1.83732	-3.70458
H	3.95534	-2.30184	-0.73661
H	5.33577	-2.93259	-2.60593

Adduct XIXb

1 1

C	1.78442	-0.43386	-1.82915
C	0.99985	0.59330	-2.34590
N	0.43177	1.56450	-1.60525
C	0.55776	1.46786	-0.24441
C	1.34270	0.47209	0.37701
C	1.96794	-0.48822	-0.42640
N	-0.08768	2.42180	0.53594
C	-0.52515	2.01835	1.88632
C	-0.79195	3.56418	-0.07929
N	2.73696	-1.47999	0.13085
C	-1.42086	0.79682	1.90451
C	-2.45333	0.69959	0.96902
C	-3.21318	-0.46373	0.89577
C	-2.91091	-1.54340	1.72353
C	-1.91544	-1.43326	2.69382
C	-1.18489	-0.24965	2.80162
C	0.04550	4.85809	0.10184
O	0.63579	5.20727	-1.13933
H	0.79936	0.66965	-3.42641
H	1.45112	0.44166	1.47671
H	0.39291	1.84904	2.50253
H	-1.04106	2.87080	2.36158
H	-0.94171	3.39707	-1.18101
H	-1.81203	3.67905	0.33423
H	2.83861	-1.55191	1.11622
H	3.15305	-2.19306	-0.42205
H	-2.62823	1.49982	0.26503
H	-4.01414	-0.54705	0.16030
H	-3.45190	-2.48110	1.60235
H	-1.69540	-2.27150	3.35264
H	-0.40263	-0.16044	3.55429
H	-0.59435	5.72064	0.34802
H	0.82899	4.75705	0.85880
H	1.16222	4.44955	-1.48481

O	4.48777	-5.24991	0.24591
H	4.02913	-6.17584	0.39169
H	4.73241	-5.14693	-0.76646
H	2.22535	-1.17192	-2.47467
H	5.35652	-5.20313	0.82590

Adduct XX

0 1

C	16.771969000	-24.511120000	-1.331926000
C	16.538732000	-25.779875000	-1.780298000
N	17.458988000	-26.766956000	-1.693007000
C	18.693241000	-26.559921000	-1.150496000
C	18.990715000	-25.259178000	-0.685989000
C	18.043864000	-24.234441000	-0.761614000
N	19.580874000	-27.588602000	-1.082270000
C	19.314552000	-28.964049000	-1.582462000
C	20.892123000	-27.386804000	-0.469055000
N	18.316603000	-22.988455000	-0.274420000
C	19.074480000	-29.966488000	-0.468632000
C	17.794152000	-30.116284000	0.083767000
C	17.579733000	-31.020318000	1.123336000
C	18.634553000	-31.789310000	1.620715000
C	19.906135000	-31.663788000	1.061859000
C	20.120858000	-30.759737000	0.019824000
C	21.931042000	-26.845053000	-1.454479000
O	23.129465000	-26.665358000	-0.714720000
H	16.009195000	-23.745827000	-1.411138000
H	15.609061000	-26.101790000	-2.238348000
H	19.962948000	-25.041247000	-0.267440000
H	18.464963000	-28.957370000	-2.268777000
H	20.194128000	-29.258861000	-2.165374000
H	20.820695000	-26.720806000	0.394765000
H	21.233689000	-28.346363000	-0.078024000
H	19.262266000	-22.745395000	-0.026647000
H	17.707960000	-22.222282000	-0.512583000
H	16.961673000	-29.560983000	-0.338829000
H	16.581510000	-31.135756000	1.535431000
H	18.461809000	-32.494978000	2.428281000
H	20.727592000	-32.272937000	1.427920000
H	21.108605000	-30.686367000	-0.430472000
H	22.059364000	-27.561579000	-2.279939000
H	21.575916000	-25.900686000	-1.895116000
H	23.834623000	-26.428008000	-1.329376000
H	17.069581000	-27.704065000	-2.118839000
Cl	16.001884000	-28.932057000	-2.929200000

Adduct XXI

0 1

C	3.459923000	-1.930862000	0.192286000
C	2.359921000	-2.251496000	0.935498000

N	1.274178000	-1.449523000	1.018120000
C	1.204831000	-0.255299000	0.362706000
C	2.324464000	0.130803000	-0.407717000
C	3.447283000	-0.694677000	-0.508587000
N	0.086985000	0.511764000	0.475568000
C	-1.101897000	0.150866000	1.293660000
C	-0.011575000	1.776305000	-0.250667000
N	4.511810000	-0.340854000	-1.286926000
C	-2.287678000	-0.293005000	0.456925000
C	-2.398908000	-1.627579000	0.040505000
C	-3.476026000	-2.029125000	-0.748508000
C	-4.457139000	-1.109559000	-1.126897000
C	-4.367037000	0.214413000	-0.697949000
C	-3.289433000	0.616485000	0.093471000
C	0.605003000	2.949809000	0.515212000
O	0.470654000	4.089103000	-0.321274000
H	4.310254000	-2.600372000	0.142667000
H	2.262503000	-3.163572000	1.515457000
H	2.324839000	1.077819000	-0.928344000
H	-0.841065000	-0.626995000	2.014590000
H	-1.366203000	1.044712000	1.869445000
H	0.450631000	1.696649000	-1.238007000
H	-1.067849000	1.980638000	-0.431856000
H	4.577652000	0.598165000	-1.645611000
H	5.379551000	-0.843174000	-1.192657000
H	-1.667976000	-2.355874000	0.380071000
H	-3.557322000	-3.067773000	-1.055431000
H	-5.296388000	-1.427931000	-1.738674000
H	-5.136450000	0.931433000	-0.969503000
H	-3.239428000	1.643900000	0.448018000
H	0.078457000	3.077083000	1.473238000
H	1.659197000	2.734970000	0.748928000
H	0.751098000	4.867285000	0.175887000
H	0.509217000	-1.892553000	1.673678000
Cl	-0.369998000	-3.001947000	2.814191000

Adduct XXII

0 1

C	3.460773000	-1.929249000	0.192552000
C	2.360674000	-2.250765000	0.935237000
N	1.274521000	-1.449349000	1.017879000
C	1.204825000	-0.254843000	0.363018000
C	2.324534000	0.132163000	-0.406842000
C	3.447780000	-0.692734000	-0.507732000
N	0.086577000	0.511634000	0.475873000
C	-1.102418000	0.149761000	1.293370000
C	-0.012344000	1.776476000	-0.249789000
N	4.512399000	-0.338030000	-1.285545000
C	-2.287687000	-0.294281000	0.456000000
C	-2.398104000	-1.628703000	0.038874000

C	-3.474746000	-2.030392000	-0.750715000
C	-4.456186000	-1.111126000	-1.128988000
C	-4.366894000	0.212676000	-0.699348000
C	-3.289767000	0.614890000	0.092650000
C	0.603408000	2.949908000	0.516864000
O	0.468782000	4.089542000	-0.319113000
H	4.311440000	-2.598330000	0.142897000
H	2.263495000	-3.163167000	1.514724000
H	2.324632000	1.079429000	-0.927015000
H	-0.841462000	-0.628323000	2.014015000
H	-1.367362000	1.043199000	1.869496000
H	0.450236000	1.697517000	-1.237009000
H	-1.068654000	1.980388000	-0.431239000
H	4.577915000	0.601194000	-1.643751000
H	5.380348000	-0.839980000	-1.191223000
H	-1.666927000	-2.356801000	0.378331000
H	-3.555416000	-3.068926000	-1.058185000
H	-5.295063000	-1.429611000	-1.741217000
H	-5.136569000	0.929448000	-0.970814000
H	-3.240398000	1.642154000	0.447726000
H	0.076481000	3.076460000	1.474777000
H	1.657629000	2.735469000	0.750828000
H	0.748687000	4.867619000	0.178517000
H	0.509548000	-1.893064000	1.672961000
Cl	-0.369526000	-3.003435000	2.812630000

Adduct XXIV

0 1

C	3.459860000	-1.930941000	0.192585000
C	2.359668000	-2.251785000	0.935423000
N	1.273911000	-1.449827000	1.018008000
C	1.204741000	-0.255408000	0.362932000
C	2.324572000	0.130912000	-0.407095000
C	3.447409000	-0.694549000	-0.507926000
N	0.086875000	0.511634000	0.475740000
C	-1.102219000	0.150502000	1.293421000
C	-0.011490000	1.776390000	-0.250146000
N	4.512135000	-0.340507000	-1.285894000
C	-2.287793000	-0.293099000	0.456250000
C	-2.398923000	-1.627542000	0.039384000
C	-3.475847000	-2.028837000	-0.750023000
C	-4.456862000	-1.109148000	-1.128368000
C	-4.366862000	0.214689000	-0.698983000
C	-3.289452000	0.616509000	0.092832000
C	0.604906000	2.949662000	0.516235000
O	0.470776000	4.089206000	-0.319946000
H	4.310198000	-2.600445000	0.142982000
H	2.262094000	-3.164033000	1.515085000
H	2.325087000	1.078082000	-0.927442000
H	-0.841579000	-0.627582000	2.014180000

H	-1.366660000	1.044176000	1.869412000
H	0.450964000	1.697022000	-1.237393000
H	-1.067716000	1.980787000	-0.431541000
H	4.578076000	0.598619000	-1.644280000
H	5.379848000	-0.842860000	-1.191551000
H	-1.668078000	-2.355945000	0.378903000
H	-3.557070000	-3.067387000	-1.057292000
H	-5.295960000	-1.427325000	-1.740454000
H	-5.136203000	0.931798000	-0.970504000
H	-3.239531000	1.643813000	0.447713000
H	0.078121000	3.076656000	1.474168000
H	1.659040000	2.734745000	0.750152000
H	0.751100000	4.867238000	0.177516000
H	0.508777000	-1.893050000	1.673235000
Cl	-0.370745000	-3.002781000	2.813180000

Adduct XXV

0 1

C	16.374740000	-25.983255000	-0.931234000
C	16.817065000	-26.955651000	-1.914103000
N	17.975207000	-27.028148000	-2.468202000
C	19.006562000	-26.081899000	-2.037330000
C	18.516308000	-24.822051000	-1.259493000
C	17.181493000	-24.948544000	-0.585960000
N	20.074127000	-26.741699000	-1.280936000
C	19.635764000	-27.502218000	-0.104956000
C	21.087996000	-27.405299000	-2.106936000
N	16.905745000	-24.013587000	0.385434000
C	20.736270000	-27.696287000	0.924515000
C	21.613200000	-26.654353000	1.254583000
C	22.580842000	-26.824940000	2.243574000
C	22.685683000	-28.041020000	2.922874000
C	21.819803000	-29.085729000	2.600171000
C	20.856172000	-28.913135000	1.604708000
C	20.645614000	-28.627272000	-2.922578000
O	21.829002000	-29.127765000	-3.543416000
H	15.391045000	-26.099170000	-0.485810000
H	16.085906000	-27.710100000	-2.216660000
H	19.289595000	-24.540151000	-0.543935000
H	18.820562000	-26.937411000	0.363152000
H	19.205364000	-28.481982000	-0.362010000
H	21.506740000	-26.664977000	-2.798271000
H	21.905014000	-27.717892000	-1.449560000
H	17.330699000	-23.104575000	0.256548000
H	15.946411000	-23.963842000	0.700413000
H	21.539331000	-25.715185000	0.714627000
H	23.255556000	-26.008112000	2.484495000
H	23.440242000	-28.173939000	3.692752000
H	21.898256000	-30.038461000	3.116215000
H	20.188749000	-29.733661000	1.351972000

H	20.191846000	-29.382738000	-2.262645000
H	19.884015000	-28.340722000	-3.658155000
H	21.572273000	-29.850074000	-4.129364000
H	19.482453000	-25.710466000	-2.948735000
Cl	18.422375000	-23.353125000	-2.396931000

Adduct XXVI

0 1

C	18.061222000	-24.516315000	-2.648438000
C	19.507276000	-24.614261000	-2.567301000
N	20.197273000	-25.162100000	-1.630620000
C	19.477424000	-25.826727000	-0.542144000
C	17.966799000	-25.471577000	-0.385493000
C	17.290992000	-24.935474000	-1.613178000
N	19.637692000	-27.282625000	-0.587682000
C	19.246721000	-27.919904000	-1.850565000
C	20.888569000	-27.777664000	-0.003708000
N	15.915183000	-24.965473000	-1.605389000
C	18.909859000	-29.393357000	-1.696300000
C	18.154898000	-29.855349000	-0.609918000
C	17.801236000	-31.200098000	-0.509613000
C	18.194102000	-32.106405000	-1.497357000
C	18.948331000	-31.657802000	-2.581385000
C	19.305851000	-30.311416000	-2.675114000
C	22.195147000	-27.415593000	-0.721999000
O	23.229491000	-28.093695000	-0.010239000
H	17.614136000	-24.099783000	-3.546274000
H	20.072708000	-24.174563000	-3.393302000
H	17.446629000	-26.351995000	-0.006361000
H	18.354435000	-27.397659000	-2.216348000
H	20.001702000	-27.803709000	-2.642858000
H	20.953112000	-27.415201000	1.028676000
H	20.825698000	-28.868748000	0.050396000
H	15.476851000	-24.874474000	-0.698146000
H	15.451037000	-24.431377000	-2.327418000
H	17.863595000	-29.151759000	0.164136000
H	17.218831000	-31.542535000	0.341285000
H	17.919158000	-33.154222000	-1.418479000
H	19.266446000	-32.355664000	-3.350763000
H	19.900432000	-29.968657000	-3.518657000
H	22.155587000	-27.740691000	-1.773153000
H	22.341179000	-26.328451000	-0.724793000
H	24.074824000	-27.822605000	-0.388256000
H	19.961974000	-25.509481000	0.385021000
Cl	17.732583000	-24.214623000	0.965656000

Adduct XXVII

0 1

C	18.732572000	-23.953744000	-2.052100000
C	20.005507000	-24.201301000	-1.399713000
N	20.269984000	-25.114822000	-0.534183000

C	19.212376000	-26.068864000	-0.193484000
C	17.756868000	-25.664164000	-0.580900000
C	17.631321000	-24.656101000	-1.685154000
N	19.500900000	-27.412338000	-0.702916000
C	19.746468000	-27.498515000	-2.147318000
C	20.417375000	-28.197370000	0.130550000
N	16.394212000	-24.566092000	-2.281185000
C	19.507776000	-28.888863000	-2.711363000
C	18.403406000	-29.653824000	-2.312747000
C	18.161220000	-30.903765000	-2.880583000
C	19.018556000	-31.409156000	-3.860850000
C	20.122341000	-30.657351000	-4.262914000
C	20.365074000	-29.408674000	-3.687342000
C	21.869670000	-27.713078000	0.232529000
O	22.551952000	-28.695178000	1.011163000
H	18.681112000	-23.197670000	-2.830042000
H	20.830881000	-23.538950000	-1.674263000
H	17.201134000	-26.572612000	-0.816135000
H	19.054302000	-26.803223000	-2.637401000
H	20.754381000	-27.161375000	-2.433102000
H	20.001573000	-28.249720000	1.143328000
H	20.430584000	-29.220811000	-0.256357000
H	15.602218000	-24.778677000	-1.688689000
H	16.241376000	-23.764649000	-2.878131000
H	17.746898000	-29.265303000	-1.540014000
H	17.302548000	-31.485727000	-2.557335000
H	18.830084000	-32.383412000	-4.302483000
H	20.800257000	-31.044560000	-5.018328000
H	21.231454000	-28.830180000	-3.999268000
H	22.312882000	-27.621990000	-0.771180000
H	21.909630000	-26.719430000	0.695416000
H	23.451257000	-28.379859000	1.162151000
H	19.222235000	-26.162271000	0.895658000
Cl	16.859433000	-24.977213000	0.896570000

Adduct XXVIII

0 1

C	16.702215000	-24.622095000	-1.350059000
C	16.546048000	-25.936735000	-1.767167000
N	17.483199000	-26.883305000	-1.676116000
C	18.678753000	-26.548080000	-1.147461000
C	18.948630000	-25.229297000	-0.706974000
C	17.946393000	-24.270254000	-0.806097000
N	19.623974000	-27.548454000	-1.062007000
C	19.360071000	-28.889244000	-1.599934000
C	20.903725000	-27.348443000	-0.400290000
N	18.161584000	-22.961407000	-0.313201000
C	19.070006000	-29.931014000	-0.533643000
C	17.929445000	-29.820540000	0.275854000
C	17.659977000	-30.777725000	1.251603000

C	18.524620000	-31.861060000	1.434336000
C	19.659178000	-31.981137000	0.633675000
C	19.928342000	-31.019007000	-0.343300000
C	21.985229000	-26.801171000	-1.336653000
O	23.167577000	-26.662328000	-0.561559000
H	15.893661000	-23.904058000	-1.435234000
H	15.599569000	-26.254897000	-2.201382000
H	19.913945000	-24.953308000	-0.300458000
H	18.513159000	-28.798342000	-2.280236000
H	20.233183000	-29.202923000	-2.185271000
H	20.795552000	-26.680694000	0.459720000
H	21.238383000	-28.309044000	0.001916000
H	19.140598000	-22.693379000	-0.300258000
H	17.619106000	-22.258396000	-0.804912000
H	17.254296000	-28.983181000	0.126854000
H	16.772026000	-30.681828000	1.870079000
H	18.312528000	-32.605711000	2.196081000
H	20.337058000	-32.819179000	0.768636000
H	20.813348000	-31.118140000	-0.968028000
H	22.125974000	-27.497736000	-2.178036000
H	21.659228000	-25.839383000	-1.762297000
H	23.878596000	-26.364176000	-1.141908000
H	17.644471000	-22.747342000	1.395411000
Cl	17.287404000	-22.483548000	2.653728000

Adduct XXIX

0 1

C	16.649875000	-24.651069000	-0.605182000
C	16.718459000	-25.869450000	0.056257000
N	17.724464000	-26.740746000	-0.050304000
C	18.754585000	-26.428741000	-0.864378000
C	18.775510000	-25.218253000	-1.599424000
C	17.716695000	-24.327968000	-1.456757000
N	19.780666000	-27.346036000	-0.949284000
C	19.706797000	-28.633910000	-0.247279000
C	21.001673000	-27.073712000	-1.691341000
N	17.739716000	-23.080746000	-2.124777000
C	20.579045000	-28.706047000	0.993870000
C	20.318598000	-27.875794000	2.094426000
C	21.115723000	-27.943460000	3.235004000
C	22.185362000	-28.841707000	3.295345000
C	22.450993000	-29.672487000	2.208173000
C	21.651097000	-29.601827000	1.064587000
C	20.909142000	-27.472454000	-3.167256000
O	22.161768000	-27.155595000	-3.757600000
H	15.811279000	-23.978249000	-0.461434000
H	15.910569000	-26.166080000	0.723278000
H	19.591904000	-24.974888000	-2.268411000
H	18.661002000	-28.793889000	0.015465000
H	20.005540000	-29.424302000	-0.946823000

H	21.273374000	-26.016453000	-1.616600000
H	21.820109000	-27.630671000	-1.226056000
H	18.305555000	-23.087300000	-2.967546000
H	16.812096000	-22.727038000	-2.336186000
H	19.482168000	-27.184767000	2.049197000
H	20.901746000	-27.296827000	4.081318000
H	22.805620000	-28.892198000	4.185556000
H	23.280164000	-30.373284000	2.245724000
H	21.859981000	-30.254219000	0.219548000
H	20.681278000	-28.547421000	-3.241064000
H	20.080414000	-26.930941000	-3.649402000
H	22.145502000	-27.443153000	-4.678629000
H	18.462082000	-21.758601000	-1.143714000
Cl	18.973819000	-20.709880000	-0.496603000

Adduct XXX

0 1

C	17.033884000	-24.396810000	-1.759173000
C	17.418048000	-25.267699000	-2.769433000
N	18.294730000	-26.264407000	-2.626171000
C	18.862540000	-26.445007000	-1.415307000
C	18.553741000	-25.600821000	-0.320757000
C	17.627884000	-24.578967000	-0.501535000
N	19.759514000	-27.485706000	-1.297510000
C	20.132872000	-28.300549000	-2.460874000
C	20.342755000	-27.862780000	-0.019515000
N	17.247300000	-23.761386000	0.588917000
C	19.492569000	-29.677598000	-2.475197000
C	18.100300000	-29.812621000	-2.583521000
C	17.511960000	-31.075343000	-2.600641000
C	18.304349000	-32.223655000	-2.510647000
C	19.688666000	-32.100286000	-2.405100000
C	20.276492000	-30.832719000	-2.386474000
C	21.630489000	-27.098406000	0.301981000
O	22.083183000	-27.568848000	1.563616000
H	16.301159000	-23.616785000	-1.935993000
H	16.988255000	-25.161298000	-3.764195000
H	19.022898000	-25.731644000	0.646736000
H	19.842704000	-27.739169000	-3.349130000
H	21.224521000	-28.408150000	-2.469238000
H	19.623201000	-27.720463000	0.792382000
H	20.564665000	-28.933624000	-0.043736000
H	17.957770000	-23.699596000	1.311339000
H	16.963337000	-22.829069000	0.305306000
H	17.486948000	-28.919977000	-2.662056000
H	16.432891000	-31.166101000	-2.687469000
H	17.843160000	-33.206954000	-2.523730000
H	20.312469000	-32.986790000	-2.334177000
H	21.357510000	-30.741024000	-2.306899000
H	22.370719000	-27.280011000	-0.493009000

H	21.428702000	-26.016019000	0.316778000
H	22.921788000	-27.136613000	1.766501000
H	15.803872000	-24.372218000	1.469614000
Cl	14.737903000	-24.736458000	2.184804000

Adduct XXXI

0 1

C	17.033884000	-24.396810000	-1.759173000
C	17.418048000	-25.267699000	-2.769433000
N	18.294730000	-26.264407000	-2.626171000
C	18.862540000	-26.445007000	-1.415307000
C	18.553741000	-25.600821000	-0.320757000
C	17.627884000	-24.578967000	-0.501535000
N	19.759514000	-27.485706000	-1.297510000
C	20.132872000	-28.300549000	-2.460874000
C	20.342755000	-27.862780000	-0.019515000
N	17.247300000	-23.761386000	0.588917000
C	19.492569000	-29.677598000	-2.475197000
C	18.100300000	-29.812621000	-2.583521000
C	17.511960000	-31.075343000	-2.600641000
C	18.304349000	-32.223655000	-2.510647000
C	19.688666000	-32.100286000	-2.405100000
C	20.276492000	-30.832719000	-2.386474000
C	21.630489000	-27.098406000	0.301981000
O	22.083183000	-27.568848000	1.563616000
H	16.301159000	-23.616785000	-1.935993000
H	16.988255000	-25.161298000	-3.764195000
H	19.022898000	-25.731644000	0.646736000
H	19.842704000	-27.739169000	-3.349130000
H	21.224521000	-28.408150000	-2.469238000
H	19.623201000	-27.720463000	0.792382000
H	20.564665000	-28.933624000	-0.043736000
H	17.957770000	-23.699596000	1.311339000
H	16.963337000	-22.829069000	0.305306000
H	17.486948000	-28.919977000	-2.662056000
H	16.432891000	-31.166101000	-2.687469000
H	17.843160000	-33.206954000	-2.523730000
H	20.312469000	-32.986790000	-2.334177000
H	21.357510000	-30.741024000	-2.306899000
H	22.370719000	-27.280011000	-0.493009000
H	21.428702000	-26.016019000	0.316778000
H	22.921788000	-27.136613000	1.766501000
H	15.803872000	-24.372218000	1.469614000
Cl	14.737903000	-24.736458000	2.184804000
