# Supplementary figures and images for: Experimental and computational studies on a protonated 2-pyridinyl moiety and its switchable effect for the design of thermolytic devices
Source: PLoS One. 2018 Sep 20;13(9):e0203604. doi: 10.1371/journal.pone.0203604 (PMC6147472; doi:10.1371/journal.pone.0203604)

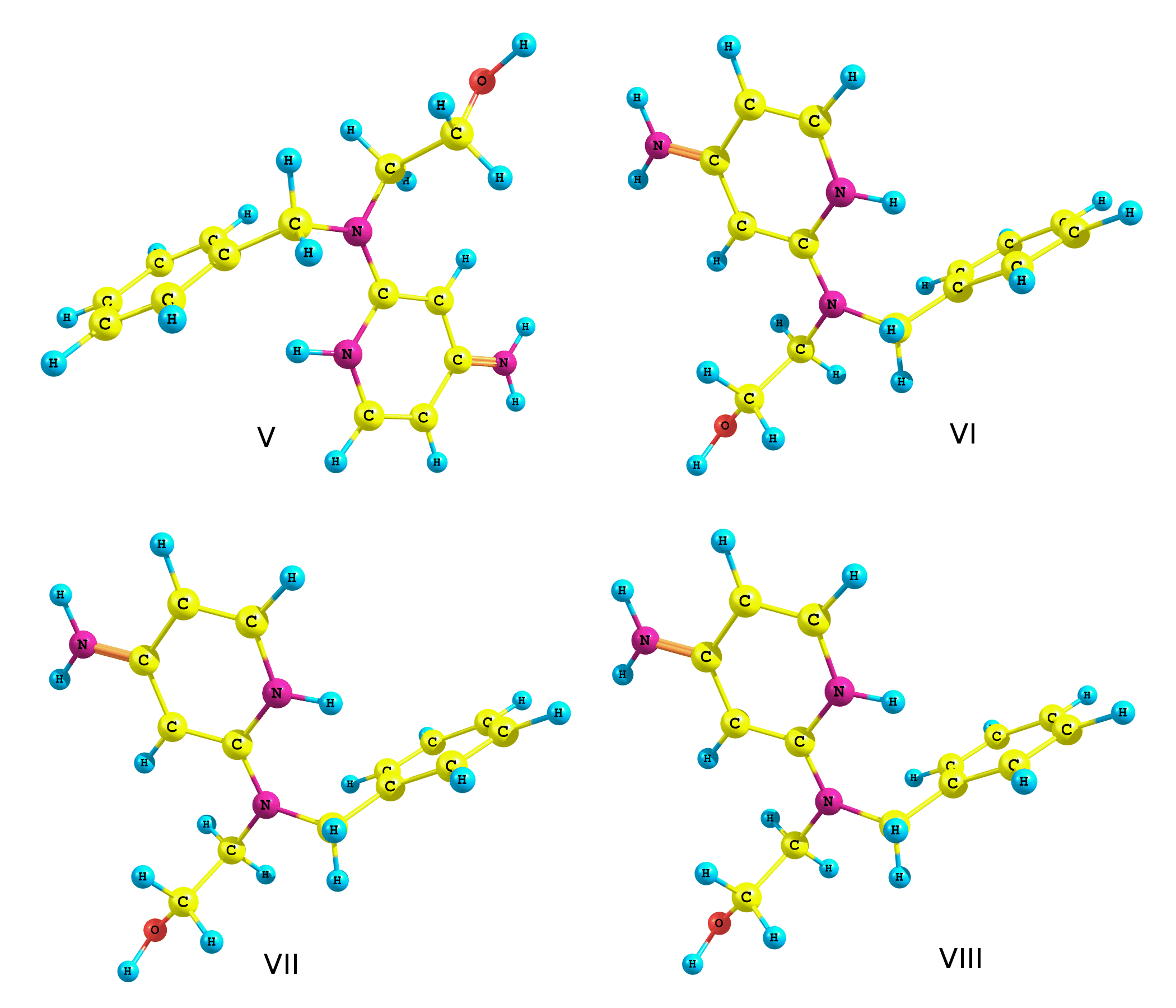

Supplement: S2 Fig — (TIFF) [file pone.0203604.s019.tiff]

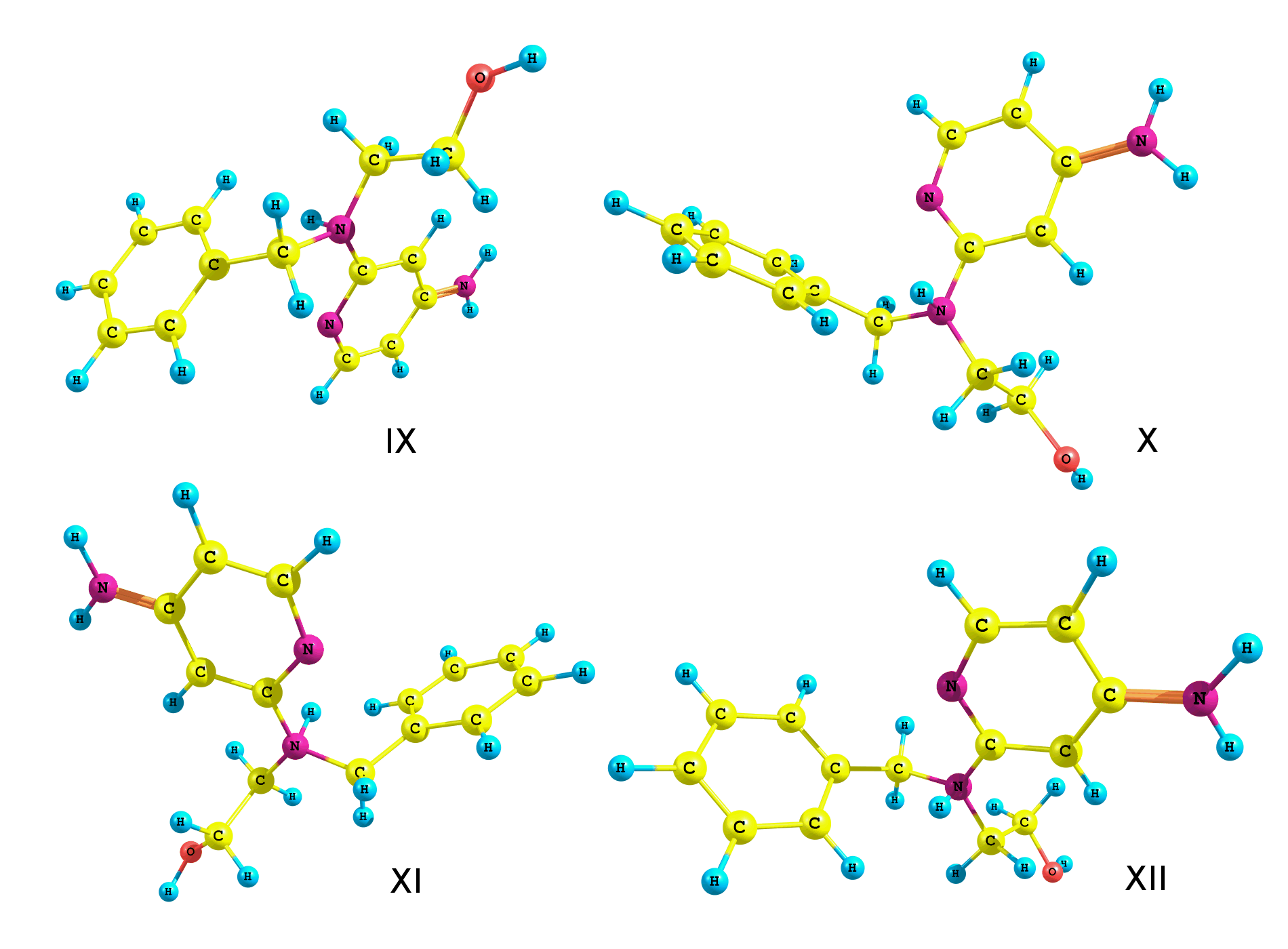

Supplement: S3 Fig — (TIFF) [file pone.0203604.s020.tiff]

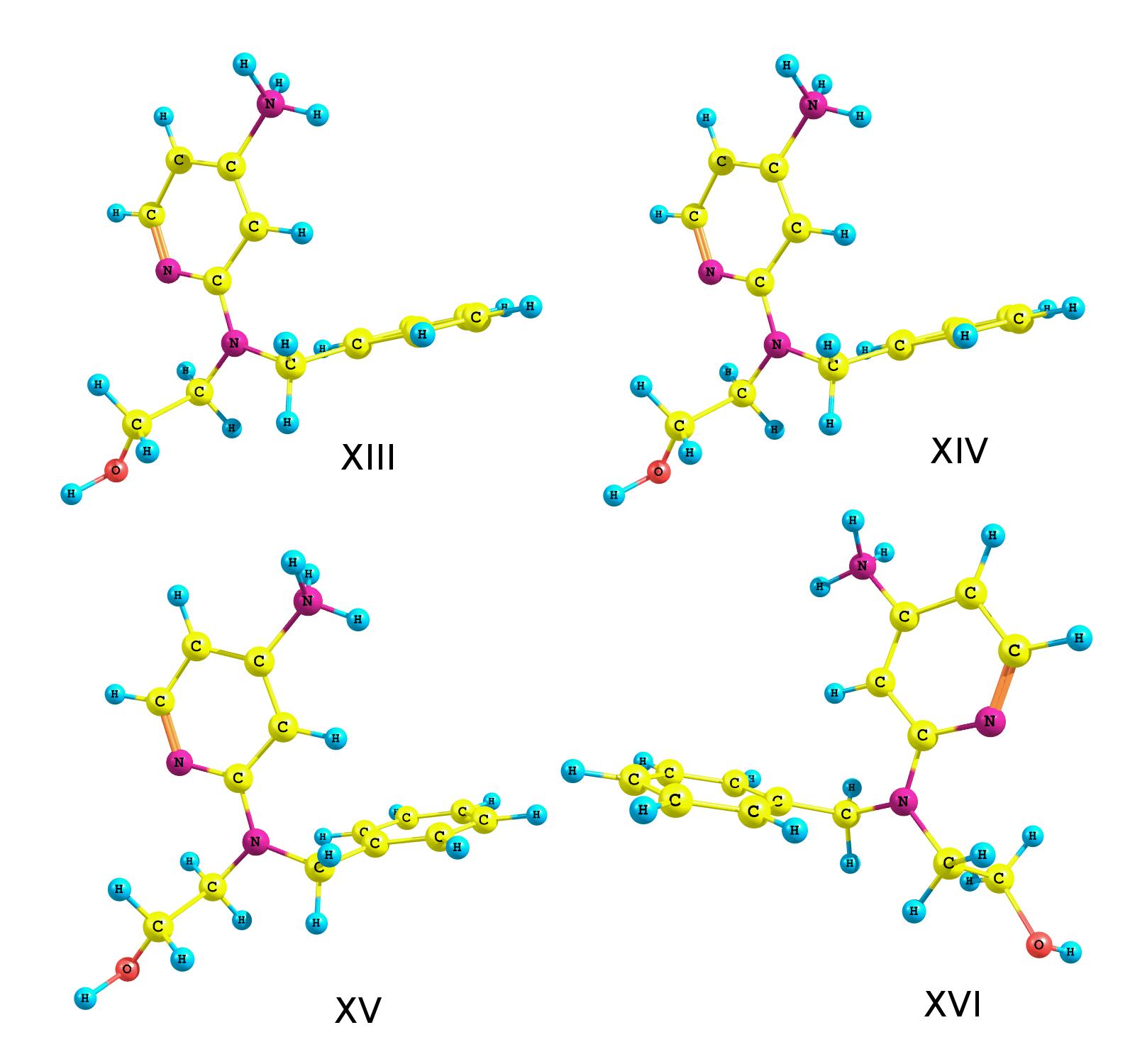

Supplement: S4 Fig — (TIFF) [file pone.0203604.s021.tiff]

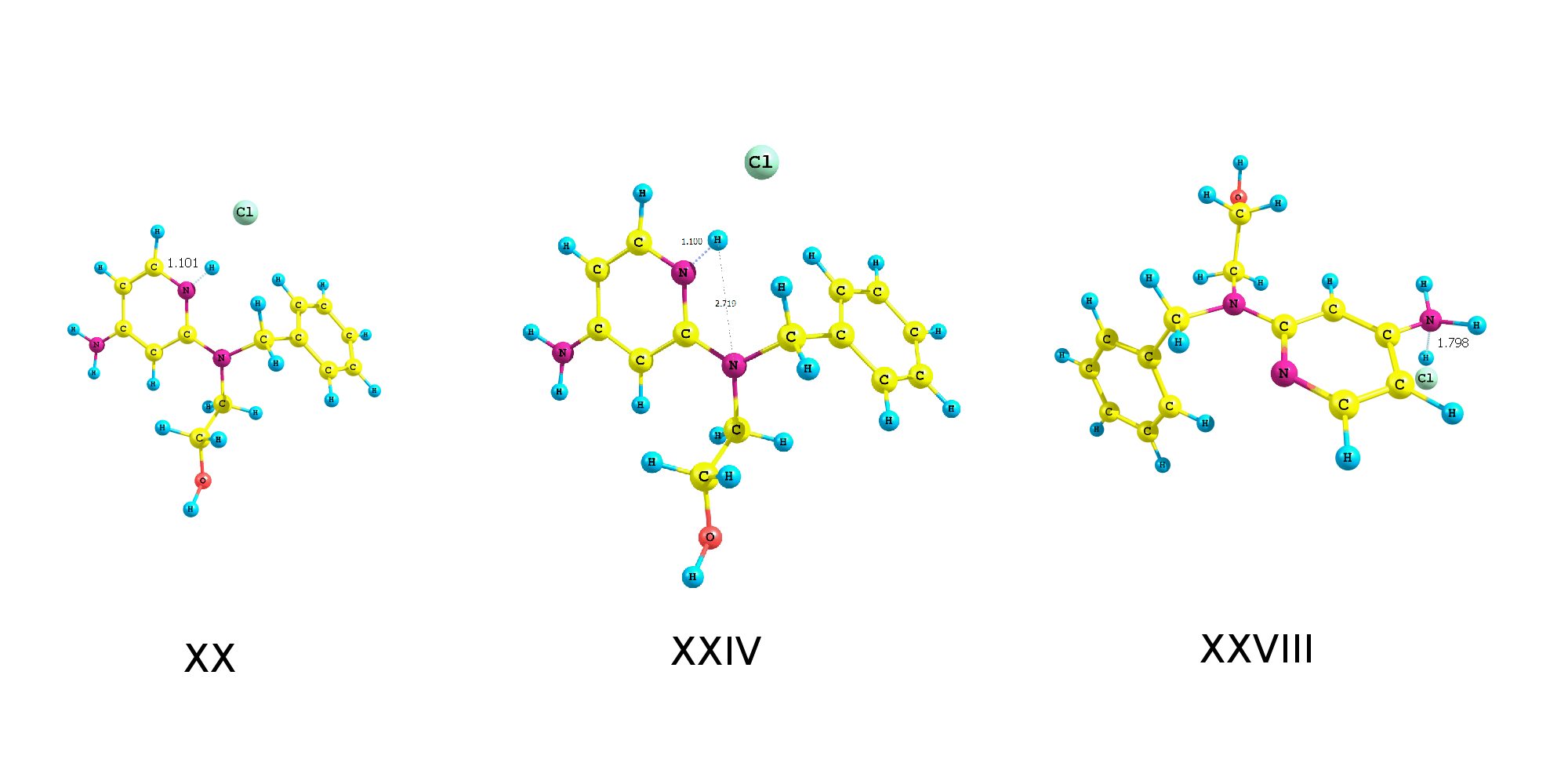

Supplement: S5 Fig — (TIFF) [file pone.0203604.s022.tiff]

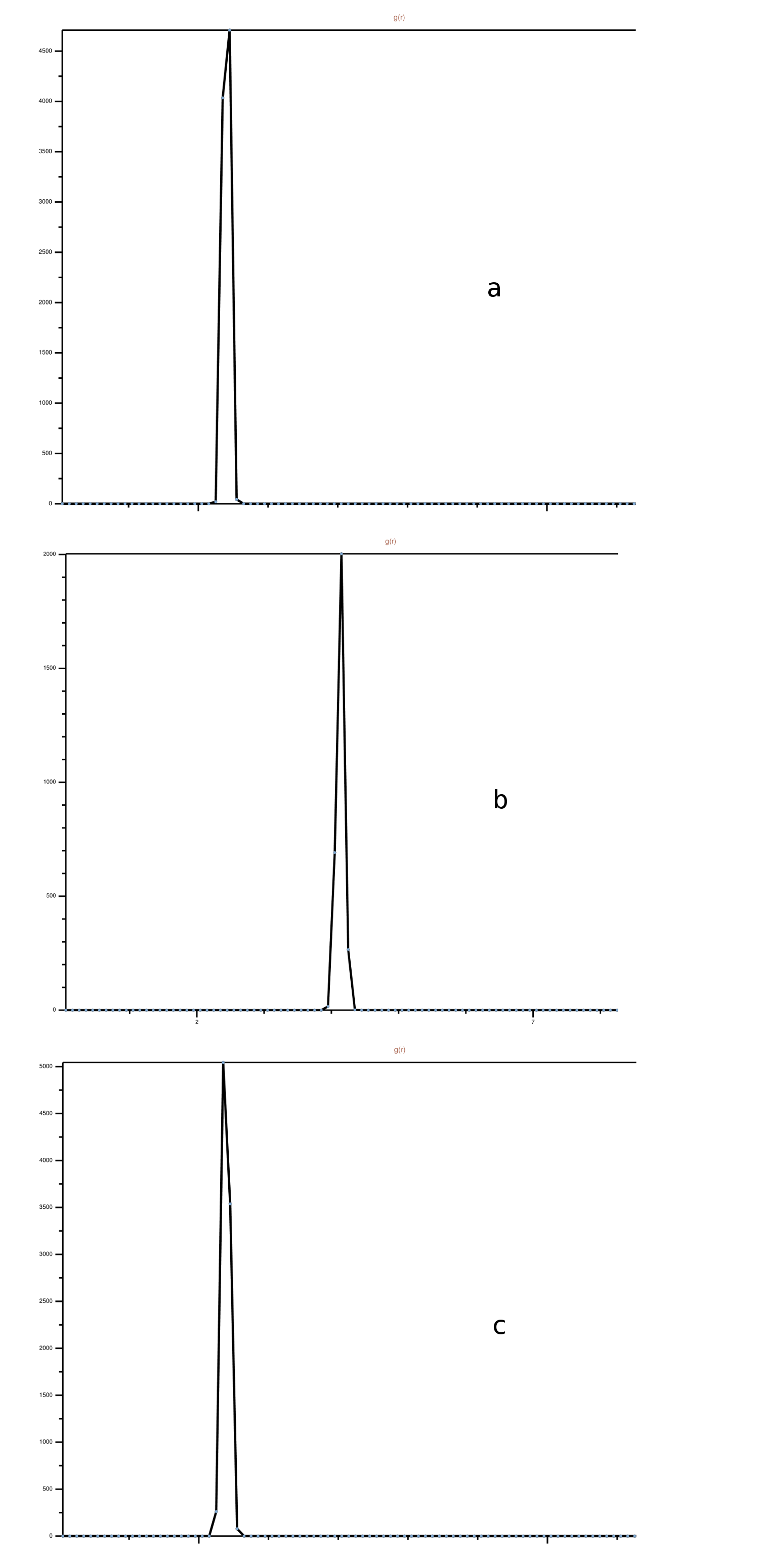

Supplement: S6 Fig — Radial distribution function of distances between the rotamer I and the water environment resulted from the classical molecular dynamics simulation (a–interaction of N1, b—interaction of N2, c—interaction of N4). (TIFF) [file pone.0203604.s023.tiff]

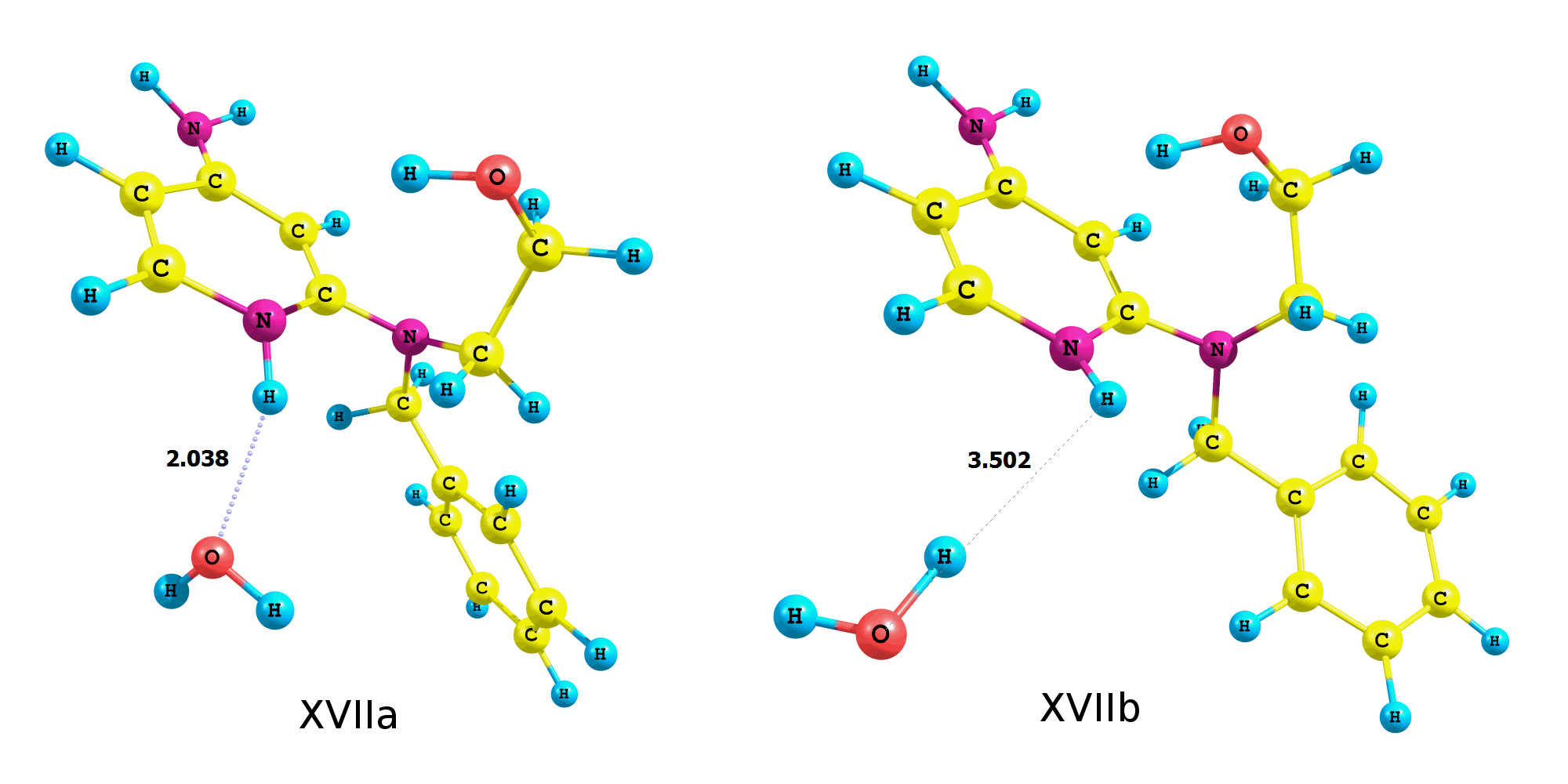

Supplement: S7 Fig — SRC simulation considering interaction of H3O+ with N1 within the adduct I–H3O+ (XVIIa–first step, XVIIb–last step); XYZ coordinates of adducts I–H3O+ (XVIIIa–first step, XVIIIb–last step) onsidering interaction of H3O+ with N2 are given below. (TIFF) [file pone.0203604.s024.tiff]

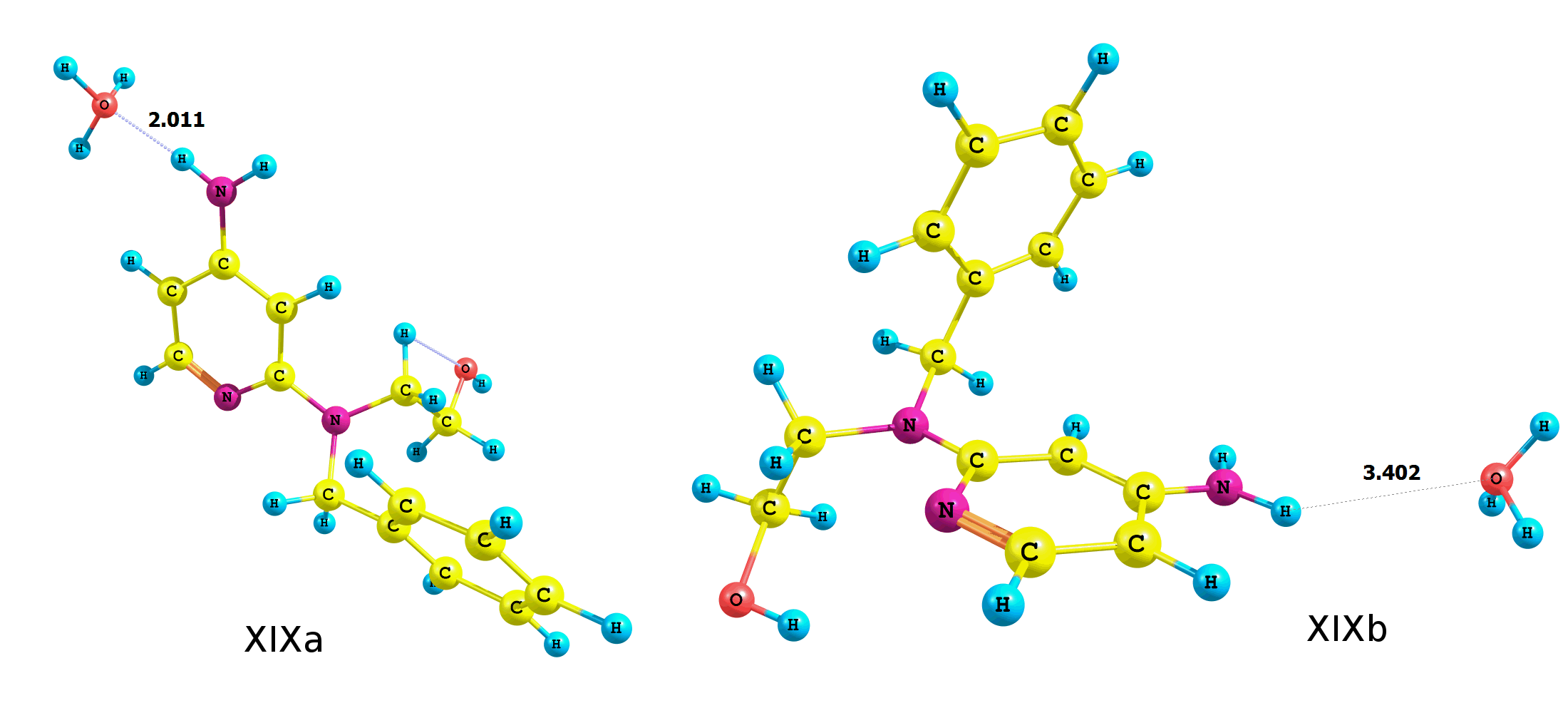

Supplement: S8 Fig — (TIFF) [file pone.0203604.s025.tiff]

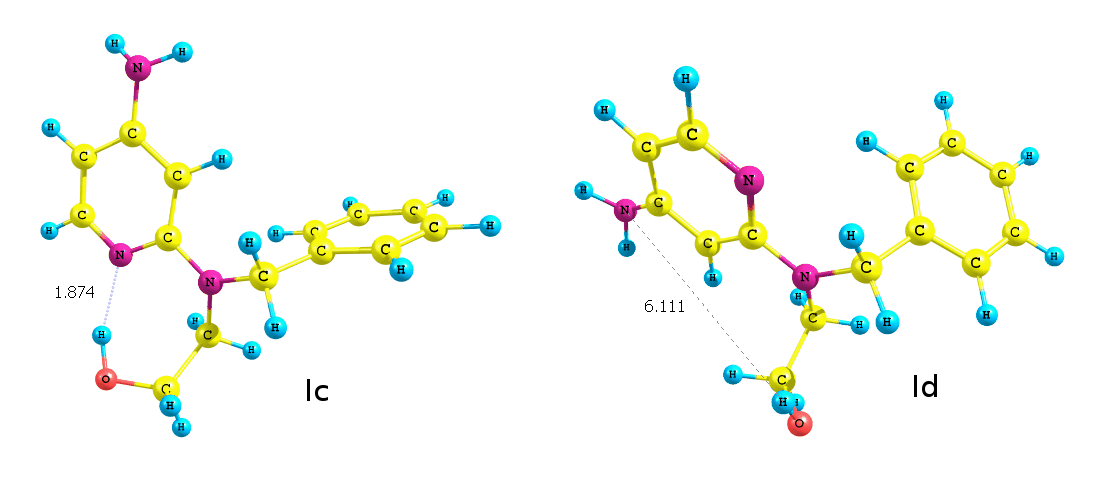

Supplement: S9 Fig — (TIFF) [file pone.0203604.s026.tiff]

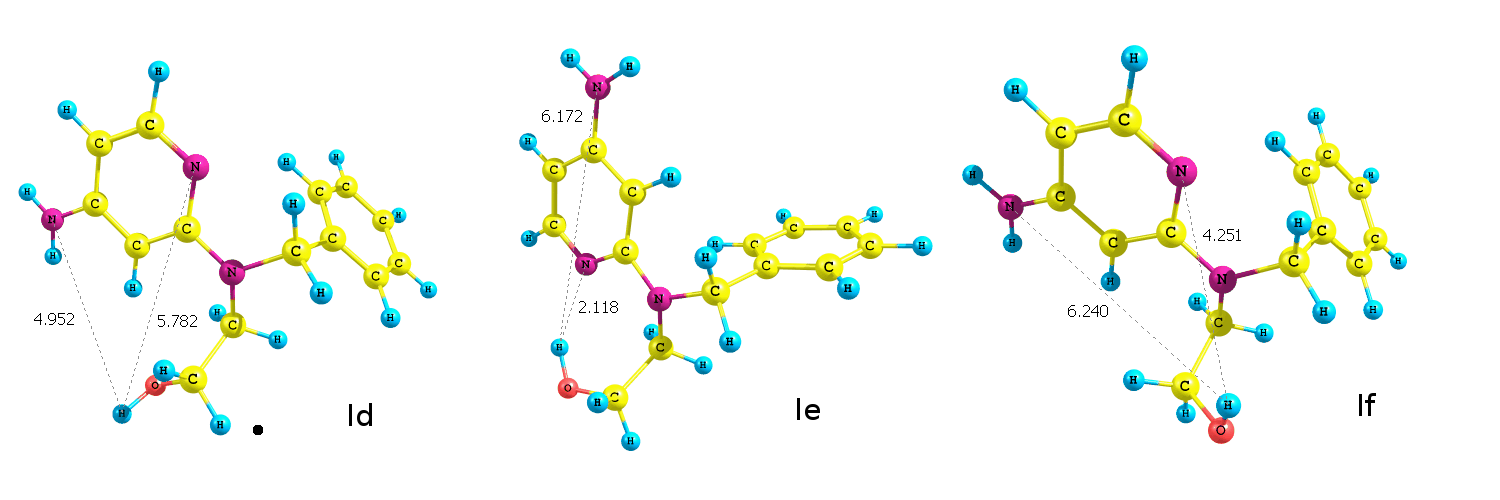

Supplement: S10 Fig — (TIFF) [file pone.0203604.s027.tiff]
